# Marked Increased Production of Acute Phase Reactants by Skeletal Muscle during Cancer Cachexia

**DOI:** 10.3390/cancers12113221

**Published:** 2020-10-31

**Authors:** Isabelle S. Massart, Geneviève Paulissen, Audrey Loumaye, Pascale Lause, Sarah A. Pötgens, Morgane M. Thibaut, Estelle Balan, Louise Deldicque, Azeddine Atfi, Edouard Louis, Damien Gruson, Laure B. Bindels, Marie-Alice Meuwis, Jean-Paul Thissen

**Affiliations:** 1 Pole of Endocrinology, Diabetes and Nutrition, Institute of Experimental and Clinical Research, Université catholique de Louvain, 1200 Brussels, Belgium; audrey.loumaye@uclouvain.be (A.L.); pascale.lause@uclouvain.be (P.L.); jeanpaul.thissen@uclouvain.be (J.-P.T.); 2 Laboratory of translational Gastroenterology, GIGA institute, Université de Liège, 4000 Liège, Belgium; genevieve.paulissen@chuliege.be (G.P.); edouard.louis@uliege.be (E.L.); marie-alice.meuwis@uliege.be (M.-A.M.); 3 Metabolism and Nutrition Research Group, Louvain Drug Research Institute, Université catholique de Louvain, 1200 Brussels, Belgium; sarah.potgens@uclouvain.be (S.A.P.); morgane.thibaut@uclouvain.be (M.M.T.); laure.bindels@uclouvain.be (L.B.B.); 4 Institute of Neuroscience, Université catholique de Louvain, 1348 Louvain-la-Neuve, Belgium; estelle.balan@uclouvain.be (E.B.); louise.deldicque@uclouvain.be (L.D.); 5 Cellular and Molecular Pathogenesis Division, Department of Pathology and Massey Cancer Center, Virginia Commonwealth University, Richmond, VA 23284, USA; Azeddine.Atfi@vcuhealth.org; 6 Centre de Recherche Saint-Antoine, Sorbonne Université, Inserm, 75012 Paris, France; 7 Hepatogastroenterology and Digestive Oncology, University Hospital of Liège, CHU, 4000 Liège, Belgium; 8 Laboratory Medicine Department, Cliniques Universitaires St-Luc, 1200 Brussels, Belgium; damien.gruson@uclouvain.be

**Keywords:** cancer, cachexia, skeletal muscle, proteomics, secretome, acute phase reactants

## Abstract

**Simple Summary:**

Muscle wasting during cancer is recognized as an independent predictor of mortality. The aim of this study was to characterize the changes in the muscle secretome associated with cancer cachexia to gain a better understanding of the mechanisms involved and to identify secreted proteins which may reflect this wasting process. Our study demonstrated that skeletal muscle is a source of several acute phase reactants during cancer cachexia that may hold the key to a cachexia-specific signature. Future work will have to determine whether some of these acute phase reactants contribute to and/or reflect the muscle atrophy caused by cancer, therefore representing potential therapeutic targets and/or biomarkers of cancer cachexia.

**Abstract:**

Loss of skeletal muscle mass in cancer cachexia is recognized as a predictor of mortality. This study aimed to characterize the changes in the muscle secretome associated with cancer cachexia to gain a better understanding of the mechanisms involved and to identify secreted proteins which may reflect this wasting process. The changes in the muscle proteome of the C26 model were investigated by label-free proteomic analysis followed by a bioinformatic analysis in order to identify potentially secreted proteins. Multiple reaction monitoring and Western blotting were used to verify the presence of candidate proteins in the circulation. Our results revealed a marked increased muscular production of several acute phase reactants (APR: Haptoglobin, Serine protease inhibitor A3N, Complement C3, Serum amyloid A-1 protein) which are released in the circulation during C26 cancer cachexia. This was confirmed in other models of cancer cachexia as well as in cancer patients. Glucocorticoids and proinflammatory cytokines are responsible for an increased production of APR by muscle cells. Finally, their muscular expressions are strongly positively correlated with body weight loss as well as the muscular induction of atrogens. Our study demonstrates therefore a marked increased production of APR by the muscle in cancer cachexia.

## 1. Introduction

Cancer cachexia is a complex metabolic syndrome characterized by loss of weight, in particular of skeletal muscle, independently of nutritional intake [1,2,3]. In addition to impairing the quality of life of patients and increasing chemotherapy toxicity, cachexia is considered to be responsible for 20% of all cancer-related deaths [4,5]. Loss of muscle mass in advanced cancer is recognized as an independent predictor of mortality. Interestingly, reversal of muscle loss leads to prolonged survival in animal models of cancer cachexia without affecting tumor growth [6,7]. This observation supports that maintaining muscle mass is per se helpful in improving survival. To date, new drugs are still under investigation to treat cancer cachexia but the lack of parameters for early diagnosis of muscle atrophy hampers the efficiency of care, which depends of the onset of therapy and its monitoring. 

Recent works indicate that skeletal muscle has a secretory function and may release into the bloodstream several proteins (named myokines), extracellular vesicles, or miRNAs. These molecules could exert biological actions in paracrine or endocrine fashion [8,9] and may reflect the metabolic processes taking place into the skeletal muscle. Investigating the muscle secretome during cancer cachexia could therefore improve our understanding on how the muscle communicates with other organs and identify potential targets directly involved in the regulation of the muscle mass. Moreover, as muscle atrophy results from disturbed protein metabolism [10], it is expected that the changes in muscle protein metabolism occurring in cachexia be reflected by the changes of several specific proteins into the bloodstream. It seems therefore plausible that changes in the levels of some of these circulating proteins released by the muscle may reflect the ongoing muscle atrophy process observed in cancer cachexia.

Therefore, we hypothesized that skeletal muscle atrophy induced by cancer is associated with specific alterations of the muscle secretome. The aims of this present experimental work were to characterize the changes in muscle proteome and to identify secretome changes during cancer cachexia, in order to better understand the mechanisms involved in this process and identify potential markers for the early diagnosis of the muscle atrophy associated with cancer cachexia. 

## 2. Results

### 2.1. C26 Colon Carcinoma Induced-Cachexia Is Associated with Profound Changes in the Skeletal Muscle Proteome

**Figure 1:**
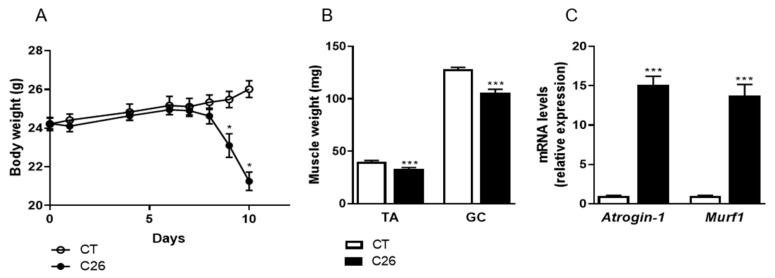
C26 colon carcinoma causes body weight loss and severe muscle atrophy. (**A**) Body weight evolution of male mice injected with C26 cells (C26) or vehicle (CT). (**B**) Tibialis anterior (TA) and gastrocnemius (GC) muscle weights from C26 and CT mice 10 days after injection. (**C**) Gene expression levels of major atrogens in GC muscle of C26 and CT mice. Data are reported as means ± SEM (*n* = 8/group). * *p* < 0.05 and *** *p* < 0.001 vs CT.

**Figure 2:**
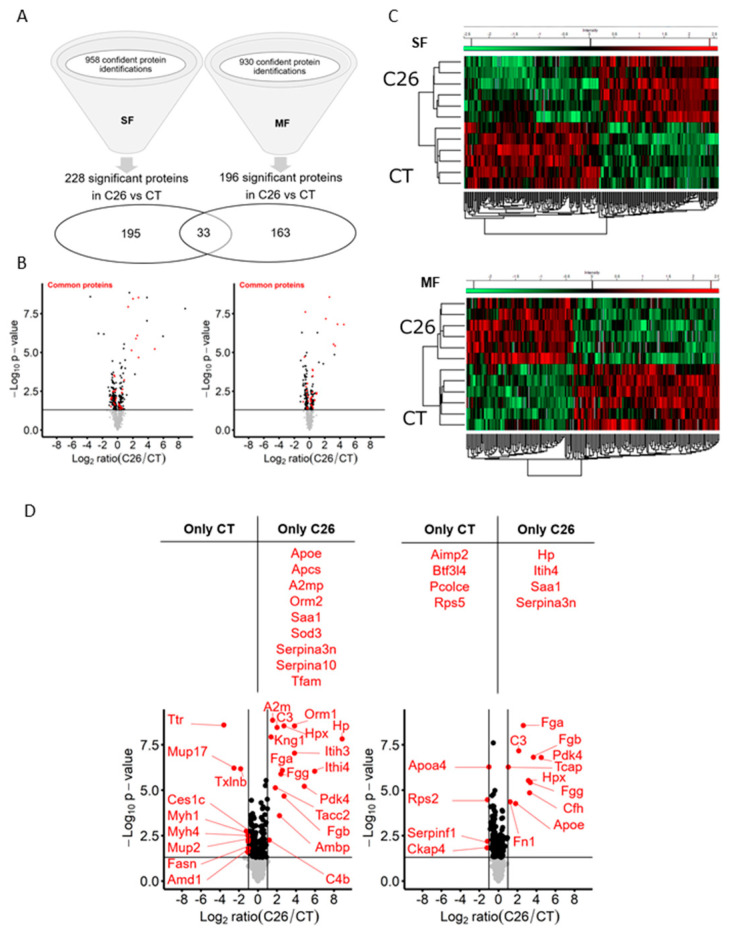
C26 cancer-induced cachexia is associated with profound changes in the skeletal muscle proteome. Global and differential proteomic analyses of gastrocnemius (GC) muscle from male mice injected with C26 cells (C26) or vehicle (CT). (**A**) Total number of confident protein identifications and differentially abundant proteins (DAPs) between C26 and CT muscle (*p* < 0.05) in the sarcoplasmic (SF) and the myofibrillar (MF) fractions. Venn diagram shows shared and unique significant DAPs in the two fractions. Full protein lists are shown in Table S1. DAPs found in the differential analysis are communicated in Table S2. (**B**) Volcano plots of the distribution of the 228 and 196 DAPs in the SF and MF, respectively. Red plots represent the 33 common proteins between the two fractions. Y-axis represents -Log10 *p*-value and x-axis represents Log2 ratio (C26/CT). The dark line shows the significance threshold limit (*p* = 0.05). (**C**) Heatmap of DAPs found in SF and MF in each mouse (*n* = 6/group) showing downregulated proteins in green and upregulated proteins in red. (**D**) Volcano plot of the proteins the most dysregulated (in red) in the skeletal muscle of C26 mice. Top: list of proteins only detected in one group (CT only or C26 only) in the SF and the MF. Bottom: DAPs between C26 and CT muscle with y-axis representing −Log10 *p*-value (threshold limit line at *p* = 0.05) and x-axis representing Log2 ratio C26/CT with vertical lines at +1 or −1 Log2 ratio (C26/CT) corresponding to a ratio C26/CT = 2 and C26/CT = 0.5, respectively.

In order to better elucidate the mechanisms of tissue wasting and, in particular, to discover proteins reflecting muscular atrophy during cancer cachexia, we carried out shotgun proteomics on skeletal muscle from C26 mice, a well-described model of cancer cachexia [11,12]. C26 mice show a marked body weight loss (Figure 1A) and profound muscle atrophy (Figure 1B) with induction of major atrogens (Figure 1C) 10 days after tumor cell injection. By using a label-free proteomic analysis on two fractions (soluble fraction or SF and myofibrillar fraction or MF) of the gastrocnemius (GC) muscle, we obtained a total of 958 and 930 confident protein identifications in the SF and the MF, respectively (FDR < 0.01; Figure 2A, Table S1). Differential analysis between C26 and CT muscles allowed the identification of 228 significant differentially abundant proteins (DAPs) in the SF and 196 in the MF (Figure 2A; Table S2) (*p*-value < 0.05). Of those, 33 proteins were common to both fractions (Figure 2A,B). Among all the proteins found with a differential distribution between C26 and CT mice, more than 50% were decreased in cachectic mice (53% in SF and 57% in MF). The heatmaps (Figure 2C) illustrate the differential distribution of the proteins highlighted with a significant difference in C26 compared to CT mice in both fractions. In addition, we included “C26 only” or “CT only” proteins that were quantified in at least five biological replicates under one condition (5/6), but could not be detected in any biological replicate under the other condition (0/6). Nine proteins were identified as “C26 only” in SF, while four proteins were “CT only” and four were “C26 only” in MF (Figure 2D). The proteins with the most extreme significant fold changes (ratio C26/CT < 0.5 or >2, see Table S2 for all ratio obtained by mathematical inverse transformation of Student’s *t*-test Difference Log2 LFQ in C26 and in CT) between C26 and CT (*p* < 0.05) are represented in red on the volcano plots of Figure 2D.

### 2.2. Mitochondrial Dysfunction, Ribosome Depletion, and Acute Phase Response Take Place in the Skeletal Muscle of C26 Mice

In order to identify the relevant biological processes involved in the muscle atrophy in C26 mice, we performed gene ontology and enrichment analyses on DAVID and STRING with the proteins showing differential distribution (up and down) in the SF and MF fractions. First, the DAVID tool allowed us to identify the top pathways altered in C26 compared to the CT muscle (Table 1). In SF and MF, the two main pathways found significantly enriched with the most abundant proteins in C26 were the acute phase reaction and the complement and coagulation cascades (*p* < 0.0001). In SF, the most significantly enriched pathway identified using the downregulated proteins was the oxidative phosphorylation with 37 counts (30.8% of total identified proteins in SF). In MF, ribosome machinery presented the highest significant enrichment (*p* < 0.0001) with 34 counts (29.6% of total identified proteins in MF). Second, the STRING protein network analysis confirmed the results from the DAVID analysis, pointing in SF, the oxidative phosphorylation (red edges) in the less abundant proteins in C26 (Figure S1A). On the other hand, in both fractions, acute phase reactants (APR) (red edges) but also complement and coagulation cascade proteins (blue edges) were identified within the proteins found more abundant in C26 compared to CT (Figure S1B,D). In MF, we also highlighted ribosomal proteins (red edges) among the less abundant proteins found in C26 compared to CT muscle (Figure S1C). 

### 2.3. Skeletal Muscle Is a Source of Several Acute Phase Reactants during Cancer Cachexia

To identify which of these DAPs are potentially secreted into the circulation during cancer cachexia, we performed a bioinformatic secretomic analysis focusing on the most differentially abundant proteins (ratio C26/CT lower than 0.5 or higher than 2; *p* < 0.05; represented on Figure 2D) and on the proteins identified as “C26 only” and “CT only” in the discovery proteomic analysis (DAPs highlighted in red on Figure 2D). This analysis allowed us to identify nine downregulated (Table 2A) and 25 upregulated proteins (Table 2B) potentially down- or up-secreted by the GC muscle from C26 mice. Among these 34 likely secreted proteins, 30 arise by the classical secretion pathway while four by the non-classical one. To verify the presence of these potentially secreted proteins in the circulation, a multiple reaction monitoring (MRM) method was elaborated specifically for targeting some of these DAPs (Supplementary methods and Tables S3 and S4). This MRM method applied on plasma samples enabled consistent validation of the differential distribution of each of the peptides belonging to the 19 proteins in the C26 model and to the 18 proteins in the BaF3 model, another model of cachexia induced by leukemia (Table S3). By comparing cachectic to CT mice, the MRM allowed the detection of specific peptides of 15 proteins increased and of one protein decreased in the circulation of both animal models of cancer cachexia. 

**Figure 3:**
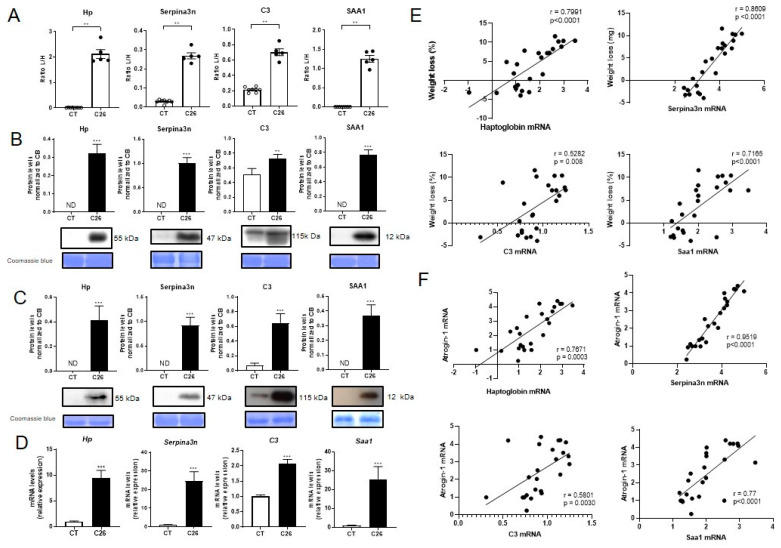
Marked production of acute phase reactants (APR) is observed in the skeletal muscle of C26 cancer-induced cachexia. (**A**) Hp, Serpina3n, C3, and SAA1 light/heavy peptide ratio measured by MRM in the plasma of C26 and CT male mice. Graphs represent the total of the two most intense transitions for one representative peptide of each protein (being a peptide without M and with the most intense mean signal observed for each protein in the C26 group). GSFPWQAK: peptide of Hp, GPGGVWAAEK: peptide of SAA1, GVFVLNK: peptide of C3, LINDYVR: peptide of Serpina3n. (**B**) Hp, Serpina3n, C3, and SAA1 protein levels measured by Western blot in the serum of C26 and CT mice. Coomassie blue staining was used as loading control. Hp, Serpina3n, C3, and SAA1 proteins. Please find the whole western blot of Figure 3B in Figure S9 (**C**) and mRNA. Please find the whole western blot of Figure 3C in Figure S10 (**D**) levels in GC muscle of mice injected with C26 cells (C26) or vehicle (CT). Coomassie blue staining was used as loading control. (**E**) Spearman correlation between log2 transformed *Hp*, *Serpina3n*, *C3*, and *Saa1* mRNA levels in GC muscle and body weight loss (%). (**F**) Spearman correlation between log2 transformed *Hp*, *Serpina3n*, *C3*, and *Saa1* mRNA levels, and log2 transformed *Atrogin-1* mRNA in GC muscle (*n* =24). Data are reported as means ± SEM (*n* = 5–10/group). ** *p* < 0.01 and *** *p* < 0.001 vs. CT.

**Figure 4:**
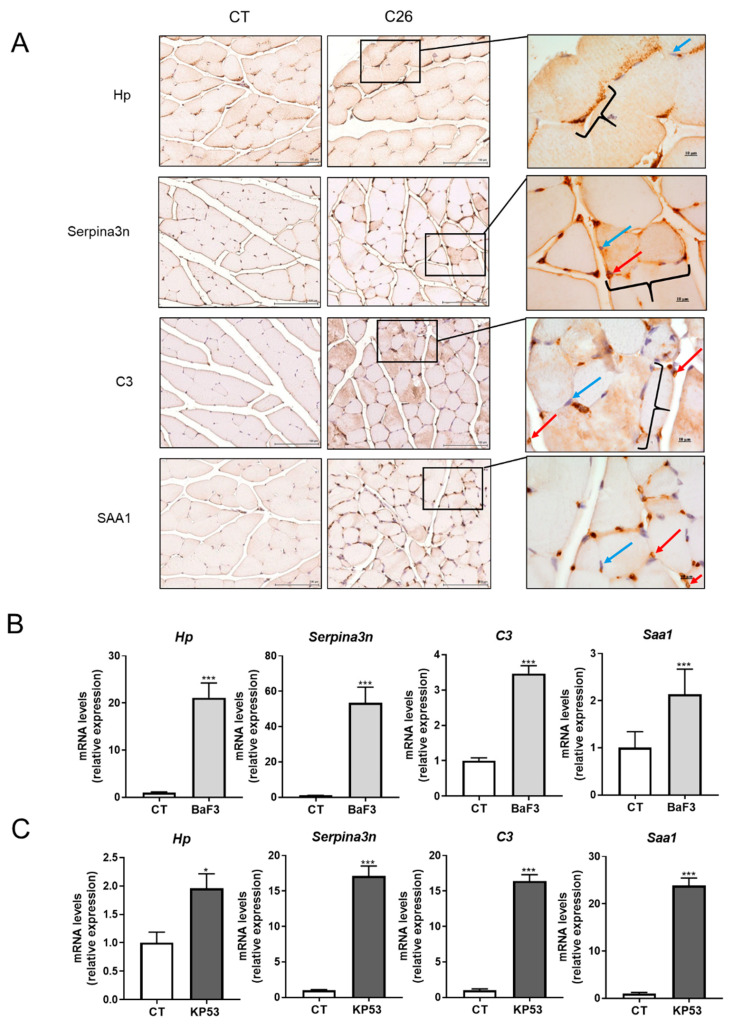
Marked production of acute phase reactants (APR) is observed in the skeletal muscle of mouse models of cancer-induced cachexia. (**A**) Immunohistochemical staining for Hp, Serpina3n, C3, and SAA1 in FFPE sections of GC muscle of C26 and CT male mice. Scale bar = 100 µm. Scale bar = 10 µm in zoom insets. Blue arrow shows nuclei located at the periphery of the cell. Red arrow shows capillaries. Bracket shows muscle fiber containing the protein of interest. (**B**) *Hp*, *Serpina3n*, *C3*, and *Saa1* mRNA relative levels in the GC muscle of female mice injected with BaF3 cells (BaF3) or vehicle (CT) and (**C**) *Hp*, *Serpina3n*, *C3*, and *Saa1* mRNA relative levels in the GC muscle of KP53 or CT male mice. Data are reported as means ± SEM (*n* = 5–10/group). * *p* < 0.05 and *** *p* < 0.001 vs. CT.

In order to investigate in more details the secretion of these proteins by the skeletal muscle during cancer cachexia, we focused on four proteins showing the highest ratio between C26/CT or being “C26 only” and after establishing a potential link with muscle atrophy by cross-referencing data of the literature [13,14,15,16,17,18]. These four proteins, which are markedly increased in the circulation of C26 mice, are Haptoglobin (Hp), Serine protease inhibitor A3N (Serpina3n), Complement C3 (C3) and Serum amyloid A-1 protein (SAA1) (Figure 3A,B). The increased abundance of these four proteins in the C26 muscle (Figure 3C) cannot be attributed only to potential blood contamination, as suggested by their profound increased mRNA expression observed in the C26 muscle (*p* < 0.0001; Figure 3D), suggesting an increased local production. Interestingly, their muscular expressions were strongly positively correlated with body weight loss as well as the muscular induction of atrogens, which can be considered as a proxy of muscle fiber cross sectional area (Figure 3E,F). The induction of muscle APR does not result from decreased food intake since 48 h fasting did not increase their muscle expression (Figure S3). Furthermore, their presence between muscle fibers, in particular in capillaries, as revealed by immunohistochemistry, suggests their secretion by the muscle (Figure 4A). Finally, the fact that these APR are predominantly present in SF compared to MF and are even more abundant in the SF of C26 than in control animals (Figure S2), suggests that these are indeed soluble and potentially secreted by skeletal muscle, in particular in C26 mice. To ensure the generalization of these results, we verified that the changes observed in cachectic muscles were not restricted to the C26 model. For that, we measured the muscle expression of the four selected proteins in two other mouse models of cancer cachexia: the BaF3 model, and the KP53 model of pancreatic ductal adenocarcinoma. Consistent with our results in C26 mice, we observed increased mRNA levels for the selected proteins in muscles originated from BaF3 (Figure 4B) and KP53 (Figure 4C) cachectic mice [19,20]. Taken together, these observations suggest that the induction of these APR is not specific to the C26 cachexia model but is more probably a generalized response of skeletal muscle to cancer cachexia.

### 2.4. Anti-IL-6 Antibody Prevents Cancer-Induced Muscular Production of Acute Phase Reactants in C26 Mice

**Figure 5:**
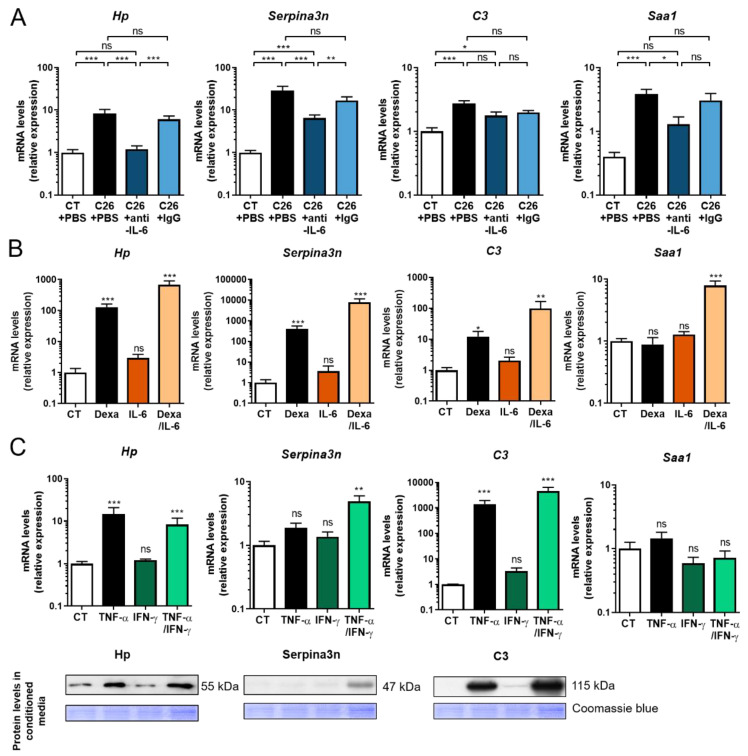
Muscular expression of APR is regulated by IL-6 in vivo and by glucocorticoids/pro inflammatory cytokines in vitro. (**A**) Anti-IL-6 antibodies prevent the increased muscular production of acute phase reactants (APR) in C26 male mice. *Hp*, *Serpina3n*, *C3*, and *Saa1* mRNA levels in the GC muscle of mice injected with C26 cells alone (C26 + PBS) or in combination with rat anti-murine IL-6 antibodies (C26 + anti-IL-6) or with rat IgG1 isotype control (C26 + IgG) or injected with vehicle alone (CT + PBS). (B-C) Glucocorticoids and proinflammatory cytokines stimulate the production of acute phase reactants (APR) by skeletal muscle cells. *Hp*, *Serpina3n*, *C3*, and *Saa1* mRNA levels in C_2_C_12_ myotubes exposed for 48 h to (**B**) IL-6 (25 ng/mL), dexamethasone (Dexa; 10^−6^ M) alone or in combination. (**C**) *Hp*, *Serpina3n*, *C3*, and *Saa1* mRNA and protein levels in C_2_C_12_ myotubes exposed for 48 h to TNF-α (5 ng/ml), IFN-γ (5 ng/mL) alone or in combination. Coomassie blue staining was used as loading control. Data are reported as means ± SEM (*n* = 3–8/group). * *p* < 0.05, ** *p* < 0.01, and *** *p* < 0.001. ns: no statistically significant when compared to CT. Please find the whole western blot of Figure 5C in the Figure S12.

In order to better understand the regulation of the muscular APR production during cancer cachexia and its association with muscular atrophy, we investigated the role of IL-6 as a mediator of their muscular induction during cancer cachexia. Indeed, circulating IL-6 levels are increased by 16-fold in our C26 model [21]. Furthermore, IL-6 is extensively known to be a key regulator of the production of liver acute phase proteins [22]. Finally, IL-6 is recognized as one of the main drivers of cachexia and muscle atrophy during cancer [21,23], as administration of neutralizing IL-6 antibodies in C26 mice prevents the muscular atrophy despite a slight increase in tumor mass [21]. As illustrated on Figure 5A, treatment with anti-IL-6 antibodies, in addition to mitigate muscle atrophy (Figure S4A), blunted the increase of three APR in the muscle (*Hp*, *Serpina3n*, *Saa1*), suggesting a potential link between their production and muscular atrophy. However, neutralization of IL-6 had no significant effect on the C3 expression. These results clearly support the role of IL-6 in the muscular induction of *Hp*, *Serpina3n*, and *Saa1* in the skeletal muscle of C26 mice. Similar results were observed in the liver of the C26 mice (Figure S4C), suggesting that the regulation of these APR by IL-6 during cancer cachexia is comparable in these two organs.

### 2.5. Glucocorticoids and Proinflammatory Cytokines Stimulate the Production of Acute Phase Reactants by Skeletal Muscle Cells

The next step was to examine whether IL-6 directly regulates these APR in muscle cells. Given the well-established role of proinflammatory cytokines and glucocorticoids in the regulation of APR production by the liver [24], we investigated the effect of IL-6, TNF-α, IFN-γ, and dexamethasone on APR expression in the mouse myoblast C_2_C_12_ cell line. First, we analyzed the role of IL-6 and glucocorticoids (Figure 5B). As expected, IL-6 did not promote the expression of *C3* in C_2_C_12_ myotubes, in agreement with our in vivo results (Figure 5A). More surprisingly, IL-6 had no effect on *Hp*, *Serpina3n*, and *Saa1* expression in C_2_C_12_ myotubes (Figure 5B) despite increased STAT3 phosphorylation (Figure S5). These observations indicate that IL-6, at least at this concentration (25 ng/mL, which is ≈100-fold higher than the in vivo circulating levels in C26 mice), does not regulate the expression of *Hp*, *Serpina3n*, *C3*, and *Saa1* in C_2_C_12_ myotubes. This could be due to the fact that C_2_C_12_ myotubes is a less relevant physiological cellular model than isolated myofibers. It is also possible that IL-6 indirectly causes the induction of these APR in the muscle. In contrast to IL-6, dexamethasone stimulated the expression of three APR (Figure 5B) and tended to potentiate the effects of IL-6 for APR production. Regarding the role of TNF-α and IFN-γ, TNF-α alone induced drastically the expression of *Hp* and *C3* while IFN-γ showed no effect (Figure 5C). Moreover, *Serpina3n* was induced exclusively by TNF-α and IFN-γ together but not separately. Finally, Western blot analysis on culture media from C_2_C_12_ exposed to these cytokines revealed that Hp, Serpina3n, and C3 were secreted by C_2_C_12_ muscle cells (Figure 5C). The role of these proinflammatory cytokines and glucocorticoids in the increased production of these APR in vivo is supported by the increased circulating levels of these mediators in cachectic mice. Taken together, these results indicate that muscle fibers themselves may participate in the increased muscle production of APR during cancer cachexia. 

### 2.6. Muscle Expression of Acute Phase Reactants Is Increased in Cancer Patients

**Figure 6:**
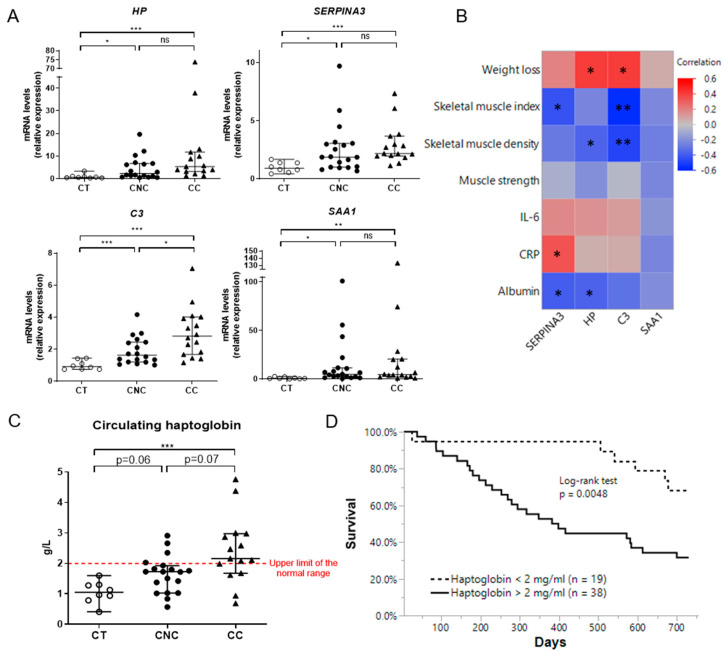
Muscle expression of acute phase reactants (APR) is increased in cancer patients and is inversely correlated with muscle index and muscle density. (**A**) *HP*, *SerpinA3*, *C3*, and *SAA1* mRNA relative levels in quadriceps biopsies of healthy subjects (CT), non-cachectic (CNC), and cachectic (CC) cancer patients. (**B**) Heatmap showing the Pearson correlation coefficients between muscular expression of four selected acute phase reactants in CNC and CC patients (*n* = 35) and, respectively, weight loss (%), skeletal muscle index (cm^2^/m^2^), skeletal muscle density (HU), muscle strength corrected with values for the 50th percentile (kg/kg) and circulating levels of IL-6, CRP, and albumin. Circulating HP concentrations are increased in CC patients and are predictive of poor survival. (**C**) Circulating levels (g/L) of HP in CT subjects, CNC and CC patients. Data are reported as median ± 95% confidence interval (CT, *n* =  8; CNC, *n* = 19; CC, *n* = 15). (**D**) Kaplan–Meier survival curves according to plasma haptoglobin levels (two groups determined with normal values range of haptoglobin: 2 mg/mL) for lung cancer patients (*n* = 57). Significance was determined by logrank test. * *p* < 0.05, ** *p* < 0.01, and *** *p* < 0.001.

To extend our murine observations to humans, we measured the expression of the four APR in skeletal muscle from cachectic and non-cachectic cancer patients in comparison to age-matched healthy subjects. As observed in Figure 6A, muscle expression of these four APR was significantly increased in cancer patients compared to control subjects (CT). However, only the *C3* expression was significantly higher (*p* < 0.05) in cachectic cancer patients compared to non-cachectic ones. Interestingly, the expression of some of these APR was negatively correlated with muscle index and/or muscle density and positively correlated with weight loss (Figure 6B). We also observed a significant increase of serum HP (Figure 6C) in CC patients in comparison with CT subjects. When the normal value threshold is applied to assess for frequency distribution among CNC and CC patients, a significantly higher number of cachectic patients show increased serum level of HP. Interestingly, HP circulating levels are correlated with its muscular expression in cancer patients (Figure S6). Furthermore, high circulating HP levels are associated with poor survival, demonstrating the clinical relevance of our findings for human cancer cachexia (Figure 6D). 

Altogether, these observations support the conclusion that the expression of these APR is induced in the human skeletal muscle during cancer and might reflect changes in muscle mass, while the circulating levels of HP are predictive of survival.

## 3. Discussion

In this present work, we characterized the changes in muscle proteome during cancer cachexia (CC) in order to identify secreted proteins which may contribute to muscle atrophy or reflect this wasting process. By using label-free proteomic analysis of skeletal muscle, we identified a pronounced increase in the production of several acute phase reactants potentially secreted by the muscle during CC. This response has been verified in several preclinical models of cancer cachexia and in cancer patients. The induction of these APR has been highlighted in muscle cells themselves as previously described in hepatocytes. Finally, we showed that proinflammatory cytokines and glucocorticoids contribute to the induction of these APR in muscle cells.

The most striking observation that we made is a marked upregulation of several acute phase reactants and complement/coagulation proteins in the muscle during cancer cachexia. Their induction is not only marked but is also consistently observed in the two fractions studied (SF and MF), confirming that their increase is real and not due to a shift from one fraction to the other. The acute phase proteins that we identified as highly upregulated are potentially secreted, in contrast to the proteins that are decreased in abundance, which are for most of them non-secreted. 

In order to investigate if skeletal muscle may release these proteins into the bloodstream during cancer cachexia, we first demonstrated by MRM the presence and the differential distribution of specific peptides belonging to most of these proteins in the plasma of two animal models of cancer cachexia. We then confirmed the presence of four selected APR (Hp, Serpina3n, C3, SAA1) in skeletal muscle by Western blot and immunohistochemistry. Furthermore, these APR are predominantly present in the SF compared to the MF and even more abundant in the SF of C26 than in control animals, suggesting they are indeed soluble and potentially secreted by skeletal muscle, in particular in C26 mice. The increased abundance of these proteins is due to an increased local production rather than a blood contamination, as suggested by the profound increased in mRNA expression observed in the skeletal muscle in different preclinical models of cancer cachexia. These four APR were also found upregulated in five microarray/RNA-seq studies assessing the C26 muscle [25,26,27,28,29], further demonstrating the synthesis of these proteins by the muscle. In addition, the fact that these APR are expressed and secreted by cultured myotubes indicates that myocytes themselves are probably responsible for their production during cancer cachexia. Finally, the positive correlation between the circulating HP levels and its muscle mRNA in cancer patients, supports the contribution of the skeletal muscle to the HP circulating concentrations. Altogether, our observations confirm and extend a previous observation [30] which suggests that, besides the liver, skeletal muscle may be also a source of APR. Although it is highly probable that the liver is the major contributor to the increase in circulating APR levels during cachexia, skeletal muscle, which encompasses about 40% of the body weight, must contribute also for a large part, even it is not definitely the only source.

The increased mRNA levels of the four selected APR (*Hp*, *Serpina3n*, *C3*, *Saa1*) in skeletal muscle from two other distinct preclinical models of cancer cachexia, namely BaF3 and KP53, suggest that their induction is not specific to the C26 cachexia model, but results more probably from a generalized response to cancer cachexia. More precisely, proinflammatory cytokines and glucocorticoids are probably involved in this upregulation, especially since their circulating levels are frequently elevated during cancer cachexia [31,32,33]. While cancer cachexia is often associated with anorexia, the induction of APR does not seem to result from reduced food intake, as we showed. It is worth noting that the induction of these APR has been also observed in other models of muscle diseases [17,34,35,36]. Finally, an increased expression of these APR was also observed in the muscle of cancer patients compared to age matched healthy subjects. However, only *C3* expression was significantly higher in cachectic cancer patients compared to non-cachectic ones. This absence of significant difference between non-cachectic or cachectic cancer patients for the other proteins (*HP*, *SERPINA3*, *SAA1*) is probably due to the progressive development of cachexia as a continuum and to the definition used to class cachectic and non-cachectic cancer patients that might be somehow arbitrary. Although another group has already reported increased HP in the muscle of cancer patients [37], our observation goes further by showing that circulating HP levels are increased in cancer cachectic patients and even more interestingly are associated with poor survival, demonstrating the clinical relevance of our findings for human cancer cachexia.

The rationale for the skeletal muscle to produce these APR in cancer cachexia is still unraveled. The possibility that some of them act via autocrine or paracrine actions on skeletal muscle has been considered and sometimes evidenced. Indeed, recent studies have demonstrated that SAA1 reduces myotube size via the activation of TLR2/TLR4//NF-κB p65 signaling pathway [14,38]. Therefore, such increase in SAA1 could worsen muscle atrophy during cancer cachexia. Conversely, C3 seems to have a protective role on skeletal muscle by facilitating muscle regeneration via the C3a–C3aR signaling pathway after injury through macrophage recruitment and infiltration [39]. Similarly, Hp seems also to have a protective role because Hp knock-out mice (Hp^−/−^) have been characterized by muscle atrophy and weakness due to the activation of an atrophy program [13] Moreover, a recent study has shown that *Serpina3n* overexpression had a profound protective effect on skeletal muscle by attenuating muscular dystrophy in the mdx or Sarcoglycan Delta (Sgcd)^−/−^ backgrounds [17]. 

A crucial question is to know whether skeletal muscle production of these APR reflects muscular atrophy itself or, more generally, the state of inflammation observed in cancer cachexia. Four clues suggest that these APR, although associated with inflammation, may also reflect muscle atrophy. First, the absence of a reduction in myotube size in response to IL-6 in vitro (Figure S7) should be compared with the absence of induction of APR in response to this cytokine, despite enhancement of STAT-3 phosphorylation (Figure S5), as opposed to dexamethasone which causes both the reduced myotube size and the APR induction (except for *Saa1*). Second, as illustrated with the anti-IL-6 experiment, neutralization of IL-6 abolishes both the muscle atrophy and the APR induction in C26 mice. Third, the muscle expression of APR is positively correlated with body weight loss and muscular induction of atrogens in C26 mice. Finally, muscle atrophy caused by glucocorticoids or streptozotocin-induced diabetes has been also associated with increased *Serpina3n* expression, one of these APR, despite the lack of inflammation in these conditions [35]. Taken together, these observations suggest that APR induction may parallel or even precede the development of muscle atrophy and could be independent of inflammation. As the APR synthesis requires amino acids, it is probable that their synthesis is achieved to the detriment of the synthesis of other proteins. This shift of amino acids toward the APR synthesis may therefore contribute to decrease the muscle mass. Unlike contractile proteins, APR are rich in aromatic amino acids, and this may lead to an excessive muscle protein breakdown to provide an adequate amount of specific amino acids for their synthesis [40]. Furthermore, in cancer patients, the muscle expression of some APR is negatively correlated with muscle index and/or muscle density reinforcing the hypothesis that their production is closely associated to muscle atrophy. Therefore, the production of APR might reflect the process of muscle atrophy during cancer cachexia.

Besides revealing the production of several APR by the muscle, our analysis also pinpointed alterations of several molecular pathways that may contribute to the muscle atrophy process in cancer cachexia. Among the main changes, we observed a marked depletion of mitochondrial proteins (belonging to complexes I, II, and IV and cytochrome C), an observation already reported in cancer cachexia both in preclinical models and in humans [41,42,43,44,45,46]. Interestingly, our analysis provides also the evidence of a marked decrease of ribosomal proteins belonging to 60S and 40S ribosomal subunits in the cachectic muscle. To our best knowledge, the decrease of ribosome machinery proteins has never been reported previously in cancer cachexia. The decrease in ribosome machinery that we observed in muscle fits perfectly with the decreased rRNA content [40] and Akt/mTOR activity [43,47] which have been previously described in the muscle of C26 mice. Altogether, these results suggest that muscle wasting during cancer cachexia could not only be due to an increasing catabolism through autophagy- or proteasome-mediated proteolysis but also to a decrease in protein synthesis. 

Our proteomic analysis was performed separately on a soluble protein fraction (SF) and a less soluble fraction containing higher levels of myofibrillar proteins (MF) in order to reduce the high dynamic range generated by highly abundant contractile proteins present in the skeletal muscle. This may restrict the detectable proteome since highly abundant proteins interfere with the detection of less abundant proteins in proteomic analyses [48]. By doing so, our muscle fractionation allowed us to detect more than 1300 unique entries in GC muscle and therefore to increase the total number of identifications in comparison with previous studies using the C26 model and assessing the soluble proteome [30,49]. Furthermore, the proteomic analysis on the insoluble protein extract (MF) revealed changes that were not identified previously by proteomics on C26 muscle (e.g., ribosome machinery). Indeed, most of the proteomic analyses performed in the context of muscle atrophy were carried out on soluble proteins extracts disregarding the changes occurring in the insoluble protein fraction [37].

## 4. Materials and Methods 

### 4.1. Animals 

#### 4.1.1. C26 Mouse Model of Cancer Cachexia 

Colon carcinoma C26 cells, provided by Dr Mario Colombo [50], were cultured in Dulbecco’s modified Eagle medium (DMEM) high glucose supplemented with 10% fetal bovine serum (PAA clone, Pasching, Austria), 100 µg/mL streptomycin, and 100 IU/mL penicillin (Gibco, Inchinnan, Scotland) at 37 °C with 5% CO_2_. After a one-week acclimatization, seven week old CD2F1 male mice (Charles River Laboratories, Wilmington, MA, USA) were injected subcutaneously in the upper flank with C26 cells (1 × 10^6^ cells in 0.1 mL saline; C26 group or C26) or with saline solution (control group or CT). Ten days after the injection of tumor cells, blood and tissue samples were harvested following anesthesia with isoflurane gas (Abbott, Ottignies, Belgium). Tissues were frozen in liquid nitrogen and stored at −80 °C until further analyses. Gastrocnemius (GC) muscles were fixed in formol. To study the kinetics of onset of cachexia, animals were necropsied 8, 9, and 10 days (*n* = 8/group) after tumor cell injection in a separate experiment. To test the role of IL-6, 300 μg of monoclonal rat anti-murine IL-6 antibody (MP5-20F3, BioXCell, Lebanon, NH, USA) or 300 μg rat IgG1 isotype control (BE0088, BioXCell) or vehicle (phosphate-buffered solution or PBS) were injected subcutaneously in C26 mice on day 7 and day 9 after tumor cell injection as previously described by Bindels et al. [21]. C26 mouse experiments were approved by the Ethical Committee for Animal care of the Health Sector of the Université Catholique de Louvain (Brussels, Belgium) in accordance with the institutional guidelines for animal care (2014/UCL/MD/010).

#### 4.1.2. BaF3 Mouse Model of Cancer Cachexia

The BaF3 mouse model and its characterization were previously reported [20]. The BaF3 cell line transfected with Bcr-Abl was a gift from Dr. K. Bhalla (MCG Cancer Center, Medical College of Georgia, Augusta, GA, USA). The BaF3 cells were maintained in RPMI1640 medium supplemented with 10% fetal bovine serum (PAA clone), 100 µg/mL streptomycin, 100 IU/mL penicillin, and 1% nonessential amino acids (Gibco) at 37 °C in humidified 5% CO_2_. After a one-week acclimatization, anesthetized five week old female BALB/c mice (Charles River Laboratories) were injected intravenously with BaF3 cells (1 × 10^6^ cells in 0.1 mL saline; BaF3 group or BaF3) or with a saline solution (control group or CT). Fourteen days after the injection of tumor cells, mice were euthanized, and blood and tissue samples were harvested then stored at −80 °C until further analyses. BaF3 mouse experiments were approved by the Ethical Committee for Animal care of the Health Sector of the Université Catholique de Louvain (Brussels, Belgium) in accordance with the institutional guidelines for animal care (2010/UCL/MD/022). Mice were housed two mice per cage with a 12 h light-dark cycle and fed normal chow diet (AO4-10, SAFE, Augy, France).

#### 4.1.3. KP53 Mouse Model of Cancer Cachexia

The pancreatic ductal adenocarcinoma (PDAC) mouse model (KP53) and its generation were previously reported [19]. Male KP53 mice were generated through successive cross breedings. Their final genotype is as following: LSL.Kras^G12D^; Trp53^fl/+^; Pdx1-Cre. All mice were maintained on a mixed C57BL/6 and FVB/N genetic background. Mice were housed with a 12 h light-dark cycle and fed a standard rodent chow diet. After 20 weeks, mice were euthanized and the development of PDAC was confirmed by hematoxylin and eosin (H&E) staining or by the ductal marker Cytokeratin 19 immunostaining on pancreatic tissue sections [19]. All animal experiments were approved by the Institutional Animal Care and Use Committee (IACUC) of the University of Mississippi Medical Center (1303A) and Virginia Commonwealth University (AD10001976) on 12 May 2014 and 26 March 2020, respectively.

### 4.2. Proteomic Discovery Analysis

#### 4.2.1. Sample Preparation, Muscle Protein Extraction, and Pre-Fractionation

Each GC muscle of CT and C26 mice (*n* = 6/group) was collected in ice-cold buffer (100 mg of tissue in 1 mL of buffer) containing 40 mM Tris-HCl (pH 7.0) with proteases inhibitors (cOmplete™, Mini Protease Inhibitor Cocktail Tablets, Roche Applied Science, Penzberg, Germany). The GC muscle was homogenized using Ultraturrax (IKA-Labortechnik, Staufen, Germany) for 6 × 10 s at 24,000 rpm at 4 °C and the homogenate was centrifuged at 10,000× *g* for 10 min to collect the supernatants with soluble proteins of the sarcoplasmic fraction (SF). The remaining pellet was resuspended in buffer containing 8 M urea and 50 mM Tris-HCl (pH 7.0) and then incubated for 1 h on ice before centrifugation at 5000× *g* for 5 min at 4 °C to collect the myofibrillar fraction (MF), containing proteins initially insoluble. This extraction protocol allowed us to obtain a MF fraction enriched in myofibrillar proteins and a SF mostly devoid of them (Figure S8). The protein concentrations of SF and MF were measured using, respectively, BiCinchoninic acid (BCA) method dosage (Pierce™ BCA Protein Assay Kit, Thermo Fisher Scientific, Waltham, MA, USA) and the Bradford method (Protein Assay Dye Reagent Concentrate, Bio-Rad, Hercules, CA, USA) according to the manufacturer’s instructions.

#### 4.2.2. Muscle Protein Extract Digestion

In total, 50 µg of protein from each sample fraction (*n* = 24, two fractions per muscle and six animals per group) were processed. The 2-D clean up kit (GE Healthcare, Chicago, IL, USA) procedure was performed on the samples, according to the manufacturer’s instructions before reduction, alkylation, and digestion steps. Prior to enzymatic digestion, the samples were resuspended in a solution of 8 M urea and 50 mM Tris-HCl (pH 8). The samples were reduced by adding dithiothreitol (DTT, Affymetrix, part of Thermo Fisher Scientific) to obtain a final concentration of 5 mM. Samples were mixed and incubated for 30 min at 37 °C. After reduction, the samples were alkylated using iodoacetamide (IAA, Sigma-Aldrich, Saint-Louis, MO, USA) at 15 mM final. Samples were mixed and incubated in the dark for 30 min at room temperature (RT). A two-step digestion was performed using Trypsin/Lys-C mix, Mass Spec Grade (Promega, Madison, WI, USA) according to manufacturer’s recommendations. Briefly, Trypsin/Lys-C mix was added to proteins extracts at enzyme to protein ratio 1:25. Samples were mixed and incubated for 3 h at 37 °C. The samples were further diluted to reduce the urea concentration to maximum 1 M and incubated overnight at 37 °C. The digestion was stopped by adding the trifluoroacetic acid (TFA, Acros Organics, part of Thermo Fisher Scientific) to 0.5% final. Finally, the samples were purified using C18 pipette tips (Pierce, Thermo Fisher Scientific) and lyophilized to dryness at RT in a speed vacuum. The pellets were stored at −20 °C until further analysis.

#### 4.2.3. Sample Protein Digests Reconditioning and Liquid Chromatography-Tandem Mass Spectrometry (LC-MS/MS) Analysis 

##### Sample Reconditioning

Prior to LC-MS/MS analysis, 2.0 µg of each protein digest was resuspended in 9 µL of 100 mM ammonium formate solution (pH 10) and spiked with the MassPREP^TM^ Digestion Standard Mixture (MPDS mix 1 (C26 samples) and 2 (CT samples); Waters, Milford, MA, USA) at 50 fmoles in ADH. This commercial mix contains yeast alcohol dehydrogenase (ADH, P00330), rabbit glycogen phosphorylase b (GPB, P00489), yeast enolase I (ENO1, P00924), and bovine serum albumin (BSA, P02769). 

##### LC-MS/MS

Purified and reconditioned protein digests were analyzed as described previously [51] on an instrumental system composed of a nanoACQUITY two-dimensional UPLC (Waters) coupled to a Q Exactive Quadrupole-Orbitrap hybrid mass spectrometer (Thermo Fisher Scientific). The 2D-UPLC separation of the peptides (2.0 µg) was obtained using a reversed-phase pH 10/reversed-phase pH 3-based separation. The first dimension separation was made at 2 μL/min (20 mM ammonium formate solution adjusted to pH 10) on an X-Bridge BEH C18, 5 μm column (300 μm × 50 mm). Three elution steps were performed at pH 10 with acetonitrile aqueous solution at 13.3%, 19%, and at 65% for fractions 1, 2, and 3, respectively. After a 1:10 dilution with acidified water, the eluted peptides were loaded on a trap column Symmetry C18, 5 μm (180 μm × 20 mm) followed by a separation on a BEH C18, 1.7 μm (75 μm × 250 mm) analytical column (Waters). Peptides were then separated using a linear gradient with solvent A [0.1% formic acid in water] and B [0.1% formic acid in acetonitrile], with a 250 nL/min flow rate; with gradient details [A/B]: 0 min, 99/1% [*v*/*v*]; 5 min, 93/7% [*v*/*v*]; 140 min, 65/35% [*v*/*v*]. The total run time was 180 min for each eluted fraction (140 min of linear gradient +40 min of cleaning and re-equilibration). The same analytical configuration (run duration: 180 min) was used for the three fractions eluted from the pH 10 column. The flow rate was constant at 250 nL/min with a linear gradient ranging from 99% water with 0.1% formic acid (*v*/*v*) to 93% after 5 min and 65% after 140 min. Solvent B was acetonitrile with 0.1% formic acid (*v*/*v*). 

##### MS/MS Data Acquisition

The mass spectrometer was operated in positive nanoESI mode with source parameters set as follows: spray voltage of 2.2 kV, capillary temperature of 270 °C, and an S-Lens RF level of 50. Data were acquired in data-dependent mode with a Top12 method. Resolution was set to 70,000 for full MS (range 400–1750 *m*/*z*) and 17,500 for MS/MS acquisitions. The automatic gain control (AGC) target and maximum injection time were, respectively, set to 1 × 10^6^ and 200 ms for MS and 1 × 10^5^ (underfill ratio of 1%) and 50 ms for MS/MS. A *m*/*z* isolation window set at 2 was used for the selection of precursors and a normalized collision energy (NCE) of 25 was used for activation of ions. A dynamic exclusion of 10 s was used. The singly charged peaks and unassigned charge states were discarded for MS/MS selection. (Each of the three UPLC fractions produced an individual raw data file after MS/MS data acquisition).

##### Raw Data Analysis

MaxQuant (version 1.5.5.1) [52] was used to manage the raw MS data files (the three raw data files obtained per sample were concatenated by MaxQuant). The following parameters for the database search and protein identification were selected: trypsin with max 2 missed cleavages, N-terminal protein acetylation and methionine oxidation were set as variable modifications and cysteine carbamidomethylation was used as fixed modification, MS/MS spectra were searched against the *mus musculus* Uniprot-release-2016_11, containing 16839 FASTA sequences using reviewed proteins. The protein and peptide false discovery rate (FDR) were both set at 0.01. Seven amino acids were the minimum peptide length and at least two peptides per protein were required for confident identification including at least one unique peptide. The precursor mass tolerance was set at 4.5 ppm. The quantification was done using the label-free quantification algorithms (LFQ) [53]. MS runs were analyzed with match between runs using the default parameter settings. The mass spectrometry proteomics data have been deposited to the ProteomeXchange Consortium via the PRIDE [54] partner repository with the dataset identifier PXD016474.

##### Differential Analysis

The Perseus program (version 1.5.5.0) was used to carry out the statistical analyses using the LFQ output generated by MaxQuant [55] and after a Log2-transformation to ensure normal distribution. Comparison of the distributions of the proteins quantified in six replicates out of six in at least one group was applied using unpaired Student’s *t*-test, with truncation based on *p*-values. Only *p* < 0.05 was considered as statistically significant. Ratios C26/CT were obtained by the mathematical inverse of the Student’s *t*-test Difference between Log2 LFQ in C26 and in CT (Table S2). We also selected proteins found only in one group and therefore below the quantitation limit for the second group. These proteins were not quantified with the instrumental system and the sample preparation protocol applied, enabling no calculated *p*-value using Perseus. These specific proteins were indeed quantified in at least five biological replicates (5/6) under one condition, but could not be detected in any biological replicate (0/6) under the other condition and were called “C26 only” or “CT only” as only providing relevant LFQ in one of the two groups (C26 or CT, respectively).

### 4.3. Gene Ontology Analysis and Identification of Enriched Pathways

DAVID (Database for Annotation, Visualization and Integrated Discovery) bioinformatics version 6.8 (https://david.ncifcrf.gov/) allowed us to evaluate the relative pathways enrichments using the proteins found significant (more or less abundant) in the comparison C26 vs. CT mice muscles, and the proteins “C26 only” or “CT only”. The analysis was performed using the SF or MF, respectively, as references background for the enrichment calculations, with *p* < 0.05 as significant threshold. STRING (Search Tool for the Retrieval of Interacting Genes/Proteins) consortium 2019 was also used (https://string-db.org/) to predict protein–protein interactions and to highlight functional enrichments submitting the significant proteins and the proteins qualified as “C26 only” or “CT only” selected by proteomics over the whole mice protein database available.

### 4.4. Targeted Proteomics Using Multiple Reaction Monitoring

In brief, the plasma samples collected for each animal of the C26 model (C26 *n* = 5, CT *n*= 8) and BaF3 model (BaF3 *n* = 7, CT *n* = 7) were used to evaluate the relative distribution in plasma of selected proteins considered as potentially secreted. A total of 20 µg of total protein extract of each mouse plasma sample were processed as described in the section “Supplementary Materials” and a fraction corresponding to 0.675 µg of protein digest was analyzed using a specific MRM methodology targeting the proteins of interest selected. We used an instrumental system coupling one dimension nanoACQUITY M-Class (Waters) on line with a triple quadrupole: Xevo TQ-S-Mass Spectrometer (Waters) equipped with nanoESI source. All the related and detailed information of sample protocols and MRM methods are available in “Supplementary Materials”. The raw data file analysis was performed using Skyline vs. 4.2.0.19072 and vs. 20.1.0.76 exclusively for the export on Panorama (https://panoramaweb.org/a1gucz.url) [56]. The data are available on ProteomeXchange under the identifier PXD019433.

### 4.5. Putative Secretomic Analysis

Proteins found to be significantly up or downregulated through proteomic differential analysis (with a ratio C26/CT lower than 0.5 or higher than 2; *p* < 0.05; refer to Table S2) together with proteins only detected in one group (CT or C26) were submitted to sequential bioinformatic secretomic analysis in order to identify differences in the muscle putative secretome between C26 and CT mice. The first step was to find prediction of signal peptide (SP) and transmembrane segment in amino acid sequences using SignalP 5.0 (www.cbs.dtu.dk/services/SignalP) and TMHMM2.0 (www.cbs.dtu.dk/services/TMHMM). Then, proteins containing SP or TM in their sequences were considered as potentially classically secreted or non-secreted, respectively. Phobius (phobius.sbc.su.se) prediction was used to confirm the presence of TM and SP. Subsequently, sequences were scanned with Prosite (prosite.expasy.org) to detect the endoplasmic retention KDEL target peptide sequence. Proteins containing KDEL sequence were considered as non-secreted. SecretomeP 2.0 Server (www.cbs.dtu.dk/services/SecretomeP) was used to detect non-classical secreted proteins. 

### 4.6. C_2_C_12_ Culture

C_2_C_12_ mouse myoblasts (American Type Culture Collection, Manassas, VA, USA) were maintained in growth medium (GM) containing DMEM high glucose with GlutaMAX (TM) supplemented with 10% fetal bovine serum, 100 µg/mL streptomycin, 100 IU/mL penicillin, and 1% nonessential amino acids (all from Life Technologies, Carlsbad, CA, USA). When the cells reached 70–80% of confluence, GM was changed with differentiation medium (DM) by replacing the 10% FBS by 2% horse serum (HS, Life Technologies) to induce myogenic differentiation. After 4 days of differentiation, cells were treated for 48 h with either TNF-α (R&D systems, Minneapolis, MN, USA) at 5 ng/mL, IFN-γ (Thermo Fisher Scientific) at 5 ng/mL, IL-6 (R&D systems) at 25 ng/mL, and Dexamethasone (Dexa, Sigma-Aldrich) at 10^−6^ M or with a combination of TNF-α and IFN-γ or IL-6 and Dexa. All the experiments were performed in triplicates in six-well plates. To analyze the secretion of proteins by C_2_C_12_ myotubes, cells were treated as described above (TNF-α, IFN-γ, IL-6, and Dexa) during 24 h in 2% HS DM followed by 24 h in serum-free conditions with the same treatments. Cells were washed three times with phosphate buffered saline before placing cells in serum-free conditions to reduce contaminating serum proteins. Serum-free conditioned media (CM) of C_2_C_12_ cells were collected and cleared by centrifugation (10 min at 300 g followed by 20 min at 2000 g) to discard dead cells. Cleared CM were subsequently concentrated using an Amicon Ultra-4 of 3 kDa cut-off spin Column (Millipore, Watford, UK) and CM aliquots were stored at −80 °C.

### 4.7. Western Blot Analyses

Proteins of GC muscles were homogenized in ice-cold buffer containing 20 mM Tris (pH 7), 270 mM sucrose, 5 mM EGTA, 1 mM EDTA, 1 mM sodium orthovanadate, 50 mM β-glycerophosphate, 5 mM sodium pyrophosphate, 50 mM sodium fluoride, 1 mM DTT, 1% *v*/*v* Triton X-100, and 10% protease inhibitor cocktail (Roche Applied Science). Homogenates were centrifuged at 10,000 g for 10 min at 4 °C, and supernatants were immediately stored at −80 °C. Muscle proteins, CM proteins, or serum proteins were resolved by sodium dodecyl sulfate-polyacrylamide gel 10% electrophoresis and transferred to PVDF membranes. Membranes were incubated overnight at 4 °C with the following primary antibodies diluted in 1% bovine serum albumin (BSA): anti-Serpina3n (1:500, AF4709, R&D Systems, anti-Haptoglobin (1:1500, LS-C404051, LifeSpan BioSciences, Seattle, WA, USA), anti-Serum Amyloid A1/A2 (1:750, AF2948, R&D Systems), and anti-C3 (1:4000, ab200999, Abcam, Cambridge, UK). Then membranes were incubated with a horseradish peroxidase (HRP) coupled to secondary antibody (Cell Signaling Technology, Danvers, MA, USA) and revelation was done using Enhanced Chemiluminescence (ECL) Western blotting Detection System Plus (GE Healthcare). The membranes were scanned with the Epson 60a and signal intensity was quantified using ImageJ software (NIH, http://imagej.nih.gov/ij). Signal intensity was normalized to whole lane of total protein loads (10–30 µg/lane) assessed by Coomassie blue staining of the membrane

### 4.8. mRNA Analysis by RT-qPCR

Total RNA was extracted from frozen muscle samples or cultured cells using the TriPure isolation reagent (Roche Applied Science) as described by the manufacturer. The cDNA was prepared by reverse transcription of 1 µg RNA using RevertAid H Minus Reverse Transcriptase (EP0451), Random Hexamer (SO142), RiboLock RNase Inhibitor (EO0381), and dNTP Mix (R0191; all from Thermo Fisher Scientific). Sybr Green® Real-time quantitative PCR was performed as previously described by Gueugneau et al. [35]. Relative mRNA levels were calculated using the comparative CT method and normalized by the expression of housekeeping genes GAPDH, cyclophilin, and RPL19. Primer sequences used for amplification during real-time qPCR are listed in Table S5 (Supplementary Materials). Primers were tested to avoid primer dimers, self-priming formation, or any unspecific amplification.

### 4.9. Immunohistochemistry

The 5 µm thick formalin fixed paraffin embedded (FFPE) were labeled with the primary and secondary antibodies and revealed with 3,3′-diaminobenzidine (DAB, Invitrogen) (details in Supplementary Materials).

### 4.10. Human Samples

Patients with colorectal or lung cancer were enrolled in a cross-sectional prospective study (ACTICA study) at the time of diagnosis or relapse between January 2012 and March 2014 at the Cliniques universitaires Saint-Luc-UCLouvain, Brussels, Belgium. The IRB protocol was approved by the Ethical Committee of the Université Catholique de Louvain on 9 May 2011 (B403201111269) and written consent was given prior to entry into the study (NCT01604642). Exclusion criteria and patient characterization, in particular the skeletal muscle and biological parameters, were reported previously [57] and in “Supplementary Materials” (Table S6). The cachectic status was determined according to the definition proposed by Fearon et al. [4], as an involuntary weight loss > 5% over the past 6 months or weight loss > 2% and body mass index (BMI) < 20 kg/m^2^ or weight loss > 2% and low muscularity. Vastus lateralis microbiopsy with a 14 Gauge true-cut biopsy needle (Bard Magnum Biopsy gun; Bard Medical, Covington, GA, USA) was performed in 35 (cancer cachectic or CC, *n*= 16 and cancer non-cachectic or CNC, *n* = 19) patients from the cohort under general anesthesia, just before the surgery for cancer. Muscle samples were cleaned of gross blood contamination and fat or fibrous tissue prior to being frozen in liquid nitrogen and stored at −80 °C until RNA extraction. RNA from vastus lateralis biopsies from age-matched healthy subjects (*n* = 8) was used as control group (CT) [58]. Serum or plasma were obtained from blood collected at the time of recruitment in standardized conditions. Circulating Haptoglobin levels were measured by clinical routine methods in our Clinical Chemistry Department using Tina-quant Haptoglobin ver.2 (Roche Diagnostics GmbH, Mannheim, Germany) and following the manufacturer’s instructions. 

### 4.11. Statistical Analysis

Results are presented as means (± SEM) for cell and animal data or medians (95% confidence interval) for human data. Numbers of individuals tested (n) are reported in the corresponding Figure legends. To assess significant differences between two groups, two-tailed, unpaired Student’s *t*-test or two-tailed Mann–Whitney–Wilcoxon test were used depending on the distribution of the quantitative variables (normal or not). When comparing more than two groups, one-way ANOVA followed by Tukey post hoc tests or multiple two-tailed Student’s *t*-tests with Bonferroni correction (if parametric) or Kruskal–Wallis global test followed by Steel–Dwass post hoc test (if nonparametric) were used according the distribution. If necessary, variables were transformed using the binary logarithm before statistical tests to meet the hypothesis of normality. All the statistical analyses were performed using the JMP Pro 14 software (SAS Institute Inc., Cary, NC, USA) and GraphPad Prism 8 software (San Diego, CA, USA). *p* <  0.05 was considered statistically significant. Pairwise Pearson correlations between APR expression in muscle and clinical or biological parameters were performed using the JMP Pro 14 software. Variables were log-transformed to meet the normality hypothesis.

## 5. Conclusions

Our study demonstrated that skeletal muscle is a source of several acute phase reactants during cancer cachexia. The increased production of these APR by muscle may hold the key to a cachexia-specific signature. Future work will have to determine whether some of these APR contribute and/or reflect the muscle atrophy caused by cancer, therefore representing potential therapeutic targets and/or biomarkers of cancer cachexia. 

## Figures and Tables

**Table 1:** The most altered pathways identified by the DAVID enrichment analyses performed on the proteins dysregulated in the skeletal muscle (SF and MF) of male C26 mice.

Fraction	Number of Proteins Submitted	Category	Term	Genes	Count	%	*p* Value	FDR
SF	Downregulated (*n* = 120)	KEGG Pathway	Oxidative phosphorylation	*Atp5f1a*, *Atp5f1b*, *Atp5f1c*, *Atp5f1d*, *Atp5me*, *Atp5mf*, *Atp5pb*, *Atp5pd*, *Atp5pf*, *Atp5po*, *Cox4i1*, *Cox5a*, *Cox5b*, *Cox6b1*, *Cyc1*, *Mtco2*, *Ndufa10*, *Ndufa11*, *Ndufa6*, *Ndufa8*, *Ndufa9*, *Ndufb10*, *Ndufb5*, *Ndufb8*, *Ndufs1*, *Ndufs2*, *Ndufs4*, *Ndufs7*, *Ndufv1*, *Ndufv2*, *Sdha*, *Sdhb*, *Uqcrb*, *Uqcrc1*, *Uqcrc2*, *Uqcrfs1*, *Uqcrh*	37	30.8	2.9 × 10^−23^	1.4 × 10^−21^
	Upregulated (*n* = 117)	KEGG Pathway	Complement and coagulation cascades	*A2m*, *F2*, *C3*, *C4b*, *Fga*, *Fgb*, *Fgg*, *Kng1*, *Serpinf2*, *Serping1*	10	8.5	2.6 × 10^−6^	4.0 × 10^−4^
		UP Keywords	Acute phase	*F2*, *Hp*, *Itih4*, *Orm1*, *Orm2*, *Serpina3n*, *Serpinf2*, *Saa1*	8	6.8	9.6 × 10^−6^	5.1 × 10^−4^
MF	Downregulated (*n* = 116)	KEGG Pathway	Ribosome	*Rpl10a*, *Rpl11*, *Rpl12*, *Rpl13*, *Rpl13a*, *Rpl14*, *Rpl18*, *Rpl19*, *Rpl30*, *Rpl4*, *Rpl6*, *Rpl7*, *Rpl7a*, *Rplp0*, *Rplp1*, *Rplp2*, *Rps10*, *Rps16*, *Rps17*, *Rps18*, *Rps19*, *Rps2*, *Rps21*, *Rps 24*, *Rps26*, *Rps28*, *Rps3*, *Rps3a1*, *Rps5*, *Rps6*, *Rps7*, *Rps8*, *Rps9*, *Rpsa*	34	29.3	5.3 × 10^−24^	3.4 × 10^−22^
	Upregulated (*n* = 88)	KEGG Pathway	Complement and coagulation cascades	*F13a1*, *C3*, *Cfh*, *Fga*, *Fgb*, *Fgg*, *Kng1*	7	8.0	5.9 × 10^−6^	8.4 × 10^−4^
		UP Keywords	Acute phase	*Fn1*, *Hp*, *Itih4*, *Serpina3n*, *Saa1*, *Stat3*	6	6.8	3.7 × 10^−8^	1.7 × 10^−6^

Category: original database where terms occur; Term: enriched terms associated with gene list; Genes: genes names implicated in the term; Count: number of genes involved in the term; %: percentage of involved genes/total genes; *p*-value: a modified Fisher exact *p*-value; FDR: false discovery rate using the Benjamini Hochberg procedure.

**Table 2:** List of proteins dysregulated and potentially secreted by the skeletal muscle of C26 mice. (**A**) Proteins the most downregulated. (**B**) Proteins the most upregulated.

Gene Name	Majority Protein ID	Protein Name	Classical Secretion (C) or Not (NC)	SF		MF	
				*p* Value	Ratio C26/CT	*p* Value	Ratio C26/CT
*Apoa4*	P06728	Apolipoprotein A-IV	C			5281 × 10^−7^	0.500
*Ces1c*	P23953	Carboxylesterase 1C	C	1779 × 10^−3^	0.426		
*Mup2*	P11589	Major urinary protein 2	C	6556 × 10^−3^	0.484		
*Mup17*	B5X0G2	Major urinary protein 17	C	6094 × 10^−7^	0.174		
*Pcolce*	Q61398	Procollagen C-endopeptidase enhancer 1	C			Only CT	
*Rps2*	P25444	40S ribosomal protein S2	NC			3339 × 10^−5^	0.448
*Rps5*	P97461	40S ribosomal protein S5	NC			Only CT	
*Serpinf1*	P97298	Pigment epithelium-derived factor	C			6618 × 10^−3^	0.446
*Ttr*	P07309	Transthyretin *	C	2578 × 10^−9^	0.083		
*Ambp*	Q07456	Protein AMBP *	C	2548 × 10^−4^	4797		
*Apcs*	P12246	Serum amyloid P-component	C	Only C26			
*Apoe*	P08226	Apolipoprotein E *	C	Only C26		5437 × 10^−5^	3505
*A2m*	Q61838	Alpha-2-macroglobulin *	C	1415 × 10^−9^	2916		
*A2mp*	Q6GQT1	Alpha-2-macroglobulin-P *	C	Only C26			
*Cfh*	P06909	Complement factor H	C			1394 × 10^−5^	9641
*C3*	P01027	Complement C3 *	C	3517 × 10^−9^	4018	6829 × 10^−8^	4402
*C4b*	P01029	Complement C4-B	C	5570 × 10^−3^	2275		
*Fga*	E9PV24	Fibrinogen alpha chain *	C	8127 × 10^−7^	5936	2736 × 10^−9^	6110
*Fgb*	Q8K0E8	Fibrinogen beta chain *	C	2127 × 10^−5^	6712	1536 × 10^−7^	12.839
*Fgg*	Q8VCM7	Fibrinogen gamma chain *	C	1298 × 10^−6^	5306	3777 × 10^−6^	10.175
*Fn1*	P11276	Fibronectin	C			4368 × 10^−5^	2340
*Hp*	Q61646	Haptoglobin *	C	1507 × 10^−8^	463.598	Only C26	
*Hpx*	Q91X72	Hemopexin *	C	2891 × 10^−9^	6647	2979 × 10^−6^	8883
*Itih3*	Q61704	Inter-alpha-trypsin inhibitor heavy chain H3 *	C	9150 × 10^−8^	14.356		
*Itih4*	A6X935	Inter alpha-trypsin inhibitor, heavy chain 4 *	C	9107 × 10^−7^	62.174	Only C26	
*Kng1*	O08677	Kininogen-1 *	C	1164 × 10^−8^	2588		
*Orm1*	Q60590	Alpha-1-acid glycoprotein 1 *	C	2897 × 10^−9^	14.330		
*Orm2*	P07361	Alpha-1-acid glycoprotein 2 *	C	Only C26			
*Pdk4*	O70571	[Pyruvate dehydrogenase (acetyl-transferring)] kinase isozyme 4, mitochondrial *	NC	6087 × 10^−6^	29.186	1593 × 10^−7^	22.703
*SAA1*	P05366	Serum amyloid A-1 protein *	C	Only C26		Only C26	
*Serpina3n*	Q91WP6	Serine protease inhibitor A3N *	C	Only C26		Only C26	
*Serpina10*	Q8R121	Protein Z-dependent protease inhibitor *	C	only C26			
*Sod3*	O09164	Extracellular superoxide dismutase [Cu-Zn]	C	Only C26			
*Tcap*	O70548	Telethonin *	NC			5359 × 10^−7^	2053

C: classical secreted proteins; NC: not classical secreted proteins; *: Proteins selected for MRM analysis at the circulating level.

## Supplementary Materials

The following are available online at https://www.mdpi.com/2072-6694/12/11/3221/s1, Figure S1: Mitochondrial dysfunction, ribosome depletion, and acute phase response take place in the skeletal muscle of C26 mice, Figure S2: Hp, Serpina3n, C3, and SAA1 content in sarcoplasmic fraction (SF) and myofibrillar fraction (MF) of gastrocnemius (GC) from mice injected with C26 cells (C26) or vehicle (CT), Figure S3: Effect of 48 h fasting on muscle APR expression, Figure S4: Anti-IL-6 antibody blunts muscle atrophy and prevents the induction of APR in the liver, Figure S5. IL-6 enhances STAT3 phosphorylation in C2C12 cells, Please find the whole western blot in the Figure S11. Figure S6: Correlation between circulating HP and HP muscle mRNA levels in cancer patients, Figure S7. Myotube size in response to glucocorticoids and IL-6 in vitro treatments, Figure S8: Enrichment of myosin heavy chains in the MF fraction. Figure S9-S12: Raw data of Western blots analysis. Table S1: List of the proteins identified and results of differential analyses comparing CT and C26 animals, Table S2: List of the proteins found differentially abundant (DAPs) between CT and C26 animals, Table S3: Illustrations of the differential distributions of the selected peptides belonging to APR proteins of interest and measured by MRM at the circulating level in C26 and BaF3 animals and their respective CT, Table S4: Technical validation on three replicates of the C26 plasma pool, Table S5: List of primers sequences used for real-time quantitative PCR analysis, Table S6: Characteristics of healthy subjects and cancer patients.
