# Supplementary material for: Marked Increased Production of Acute Phase Reactants by Skeletal Muscle during Cancer Cachexia
Source: Cancers (Basel). 2020 Oct 31;12(11):3221. doi: 10.3390/cancers12113221 (PMC7693727; doi:10.3390/cancers12113221)
Supplement: Supplementary file 1 [file cancers-12-03221-s001.zip › cancers supp DAT (ZIP)/Revised Cancers Supplementary Data Massart I .pdf]

Supplementary Materials: Marked increased production of acute phase reactants by skeletal muscle during cancer cachexia

Isabelle S. Massart, Geneviève Paulissen, Audrey Loumaye, Pascale Lause, Sarah A. Pötgens, Morgane M. Thibaut, Estelle Balan, Louise Deldicque, Azeddine Atfi, Edouard Louis, Damien Gruson, Laure B. Bindels, Marie-Alice Meuwis and Jean-Paul Thissen

Supplementary methods

Multiple Reaction Monitoring

Plasma sample collection and preparation for Multiple Reaction Monitoring

During sacrifice, blood was collected into EDTA-coated eppendorf tubes and the plasma was obtained after centrifugation at 13 000 g for 3 min at 4°C. A pool of C26 plasma was used for the development of the MRM method. The plasma samples collected during sacrifice in the two mice models C26 (C26 n = 5, CT n = 8) and BaF3 (BaF3 n = 7, CT n = 7) were treated individually to confirm the plasma relative distribution of the selected proteins. The plasma samples were stored at -80°C. The plasma protein concentrations were measured using RC-DC Protein Assay (Bio-Rad, 500/0119) according to the manufacturer's instructions.

A pool of plasma samples of the C26 animals was used as the reference sample matrix for the development of the MRM method. The C26 plasma pool (20 µg protein) spiked with 138 ng of enolase (Enolase from baker'yeast, Sigma, E6126) was processed as described in the section "*Proteomic discovery analysis",* but without any 2D-clean up. A standardized protocol of digestion was applied (Trypsin/Lys-C Mix, Mass Spec Grade, V5072, Promega, Madison, WI, USA) according to the manufacturer's instructions. Then, 3.5 µg of the resulting peptide mixtures were purified on ZipTip C18 (Thermo Fisher Scientific), dried in a vacuum centrifuge and stored at -20 C°. Before injection, dried samples (3.5 µg) were reconstituted in 41.6 µL of 0.1% formic acid and 5.2 µL of a solution containing a synthetic stable isotope-labeled peptide of the yeast Enolase ENO1 (Uniprot accession P00924) heavy AQUA peptide (VNQIGTLSESIK, ^{15}N- and ^{13}C-labeled lysine residue – HeavyPeptide ™ AQUA, Thermo Fisher Scientific) and the SpikeTides heavy peptides targeted solubilised in 5% acetonitrile.

ENO1 heavy was used to check the instrumental set-up stability across sample injections. The quantity of VNQIGTLSESIK injected was 50 fmol together with 0.675µg of reconstituted purified protein digest.

Development of the specific MRM method

Selection of the targeted peptides: For each targeted protein (see Tables 2A and 2B: proteins with *), four tryptic digestion peptides if possible quantotypic were targeted in agreement with the rules detailed previously (Lange, Picotti, Domon, & Aebersold, 2008).

Elaboration of the MRM method: We used the SpikeTides peptides corresponding to the selected peptides that were purchased heavy labelled from JPT Peptide Technologies Gmbh (Berlin, Germany). First, the labelled heavy peptides, reconstituted in 5% acetonitrile were injected with and without plasma matrix (protein digested pool of C26 mice plasma) on an Acquity M-Class UPLC (Waters, Milford, MA, USA) coupled to a Q Exactive (Thermo Fisher Scientific, Waltham, MA, USA) in nano-electrospray positive ion mode as detailed below and using the same LC conditions than these used for the downstream MRM experiments. The same instrument settings were used as for the discovery runs but with a 150 min analysed gradient. Therefore, data acquisition was adapted to this timing together with a larger MS range (from 350 to 1750 m/z). The signal of the peptides were analysed in Data Dependent Acquisition (DDA) mode and allowed to establish retention time windows used as well in the MRM experiments. The raw data were treated using Skyline vs 4.2.0.19072 and were used as library reference sample. Correct peptide identifications were controlled as well as the determination of the following parameters: retention times, elution windows, precursor masses, fragments generated and their respective signal intensities. We analysed both oxidized and non-oxidized forms of the methionine containing peptides.

LC conditions: The samples (0.675 µg of protein digest) were loaded on the trap column (Symmetry C18 5µm, 180 µm x 20 mm, Waters) in 100% solvent A (water 0.1% formic acid) during 3 min. Separation on the analytical column (HSS T3 C18 1.8 µm, 75 µm x 250 mm, Waters) was done at a flow rate of 600 nL/min, solvent A (0.1% formic acid in water) and solvent B (0.1% formic acid in acetonitrile) with linear gradient 0 min, 98% A; 5 min, 93% A; 135 min, 70% A; 150 min, 60% A. The total run time was 180 min: 150 min gradient and 30 minutes of column cleaning and reconditioning.

MRM Method refinement: We preselected the best transitions according to signal intensity and transition specificity for all the targeted peptides that were further acquired on a Xevo TQ-S mass spectrometer equipped with a nano-electrospray source (Waters) and using the same UPLC separative system (with the LC settings described above).

The Xevo TQ-S Mass Spectrometer parameters were the following: nanoESI positive polarity with capillary voltage set at 2.9 kV, Cone Voltage was set at 30, with source offset and source temperature at 50 V and 100°C respectively. The cone gas flow and the Nebulizer gas flow (both nitrogen) were set at 150 L/h and 5.0 Bar respectively. Collision gas (Argon) flow was set at 0.150 mL/min. LM1 and HM1 Resolutions were at 3.0 and 15.0 respectively, with ion Energy1 at 0.5, while LM2 and HM2 were respectively set at 3.0 and 15.0, with ion Energy2 at 0.5.

Skyline was also used to export the scheduled final MRM method allowing the detection of each peptide transitions targeted within a time window of 4 minutes.

Technical validation and finalization of the specific targeted MRM method

To finalize the targeted MRM method, we used the pool of C26 plasma and performed technical triplicates of process enabling the evaluation of the MRM method repeatability (Supplementary Table S4). The parameters of sample preparation protocol, LC and MRM data acquisition were these established during the elaboration and refinement of the MRM method and detailed in the above section. This allowed evaluation of the final refined MRM method performance to be used for the detection of 19 proteins and enolase (59 peptides including the Enolase peptide) and targeting 9 proteins with 4 peptides per protein, 5 proteins with 3 peptides per protein, 3 proteins with 2 peptides per protein and 1 protein with only 1 peptide. The majority of the transitions showed a coefficient of variation (CV) below 30%, in the C26 plasma pool replicates. However, among the 2 most intense transitions, 22 showed a CV > 30%. The CV of the VNQIGTLSESIK peptide of Enolase (Light/heavy ratio) for the two most intense transitions was < 13%.

C26 and BaF3 sample MRM data acquisition and raw data processing

The samples of each individual of the C26 and BaF3 models were processed using the standardised protocol described for the C26 plasma pool in a standardised manner. All the

samples were analysed using the scheduled final MRM method described in the above section. The Skyline files (one for the C26 and one for the BaF experiments respectively) were exported to Panorama using skyline vs 20.1.0.76 (https://panoramaweb.org/a1gucz.url) and are also referenced on ProteomeXchange under the identifier PXD019433. Results of the MRM quantification for each peptide of the APR of interest: The ratio light to heavy peptide (ratio L/H) was calculated for each transition monitored and we used the sum of the 2 most intense transitions for the peptide quantification. The heavy peptide of enolase spiked right before injection was used as an internal standard to assess the reproducibility of our digestion protocol. The CV of the light/heavy ratio for the VNQIGTLSESIK peptide of Enolase, for the two most intense transitions was 13.47 and 16.61% in the C26 and BaF experiment respectively. Oxidized and non-oxidized forms of peptides containing methionine were detected and quantified. Oxidized, non-oxidized and the sum of the two forms of these methionine containing peptides were computed and used to confirm the distribution of the peptides targeted. The result summary for the C26 and BaF3 models are provided for each peptide belonging to the APR of interest, with the mean +/- SEM in each group (Supplementary Table S3).

Immunohistochemistry

GC muscle samples were fixed in 10% formalin for 48 h and embedded in paraffin. Five µm thick formalin fixed paraffin embedded (FFPE) tissue sections were mounted on SuperFrost Plus slides (Menzel-Gläser, Braunschweig, Germany), and deparaffinized with xylene and rehydrated in a graded series of ethanol baths. Antigen-retrieval was performed for 30 min at high temperature in citrate buffer and endogenous peroxidases were inhibited with H_2O_2 3% (v/v). Then muscle sections were blocked and incubated overnight at 4°C with the following primary antibodies at 1:100 (or 1:4000 for the anti-C3 antibody): anti-Serpina3n (AF4709, R&D Systems), anti-Haptoglobin (LS-C404051, LifeSpan BioSciences), anti-Serum Amyloid A1/A2 (AF2948, R&D Systems) and anti-C3 (ab200999, Abcam). After washing, sections were incubated for 1 h, at RT with adequate secondary antibodies and the peroxidase activity was revealed with 3,3′-diaminobenzidine (DAB, Invitrogen). Muscle sections were scanned using the Leica SCN400 slide scanner (Leica Microsystems, Wetzlar, Germany).

Supplementary Figures

Figure S1

A

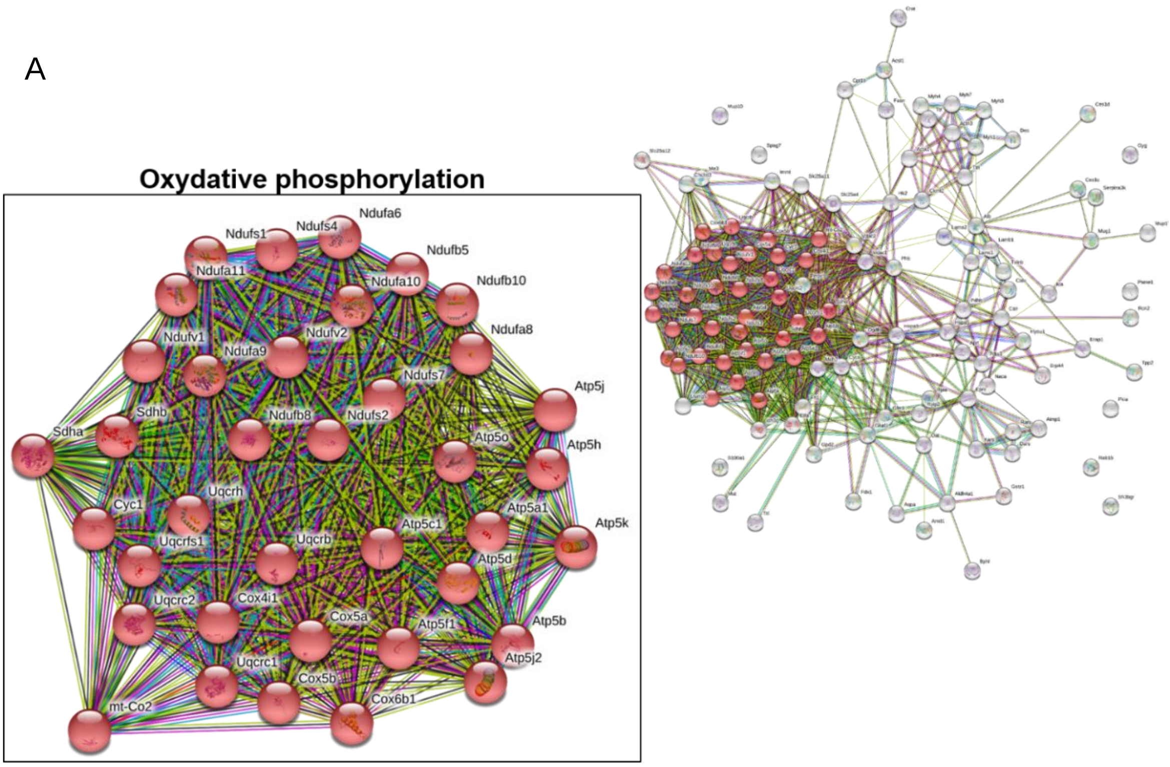

B

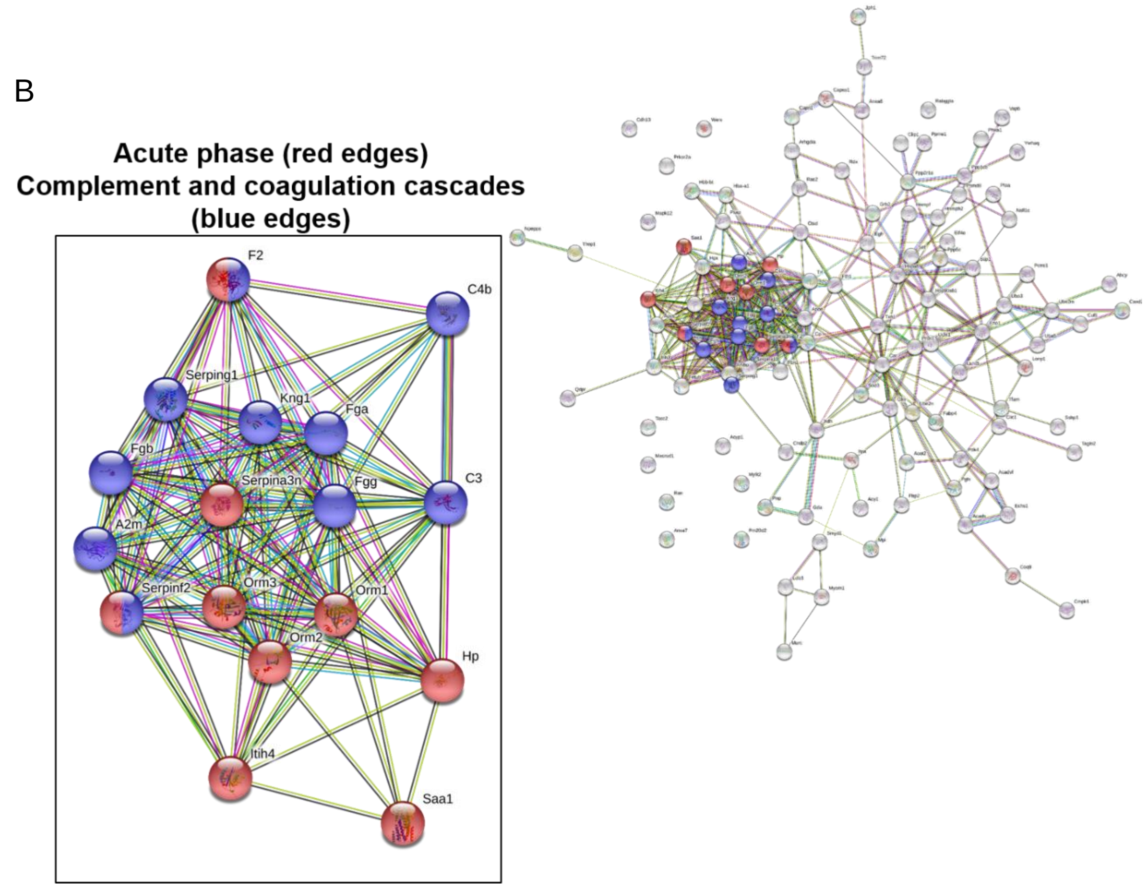

C

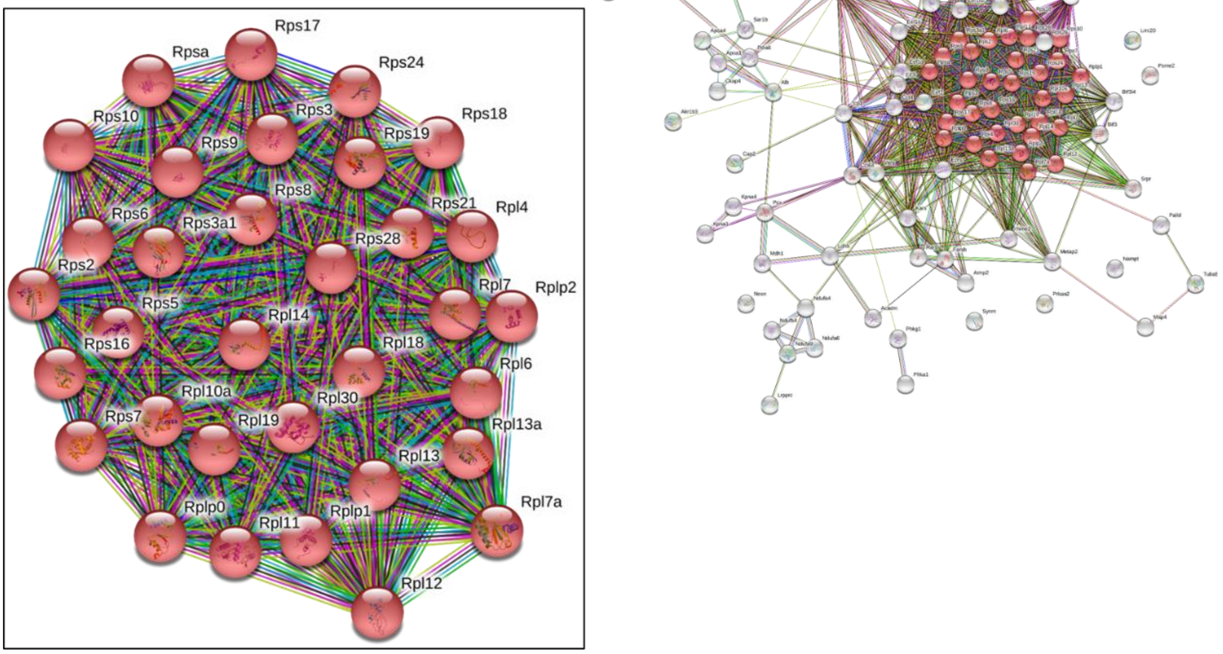
Ribosome
Rps17
Rpsa
Rps24
Rps10
Rps3
Rps18
Rps9
Rps19
Rps8
Rps6
Rps21
Rpl4
Rps3a1
Rps28
Rps2
Rpl7
Rplp2
Rps5
Rpl14
Rps16
Rpl18
Rpl6
Rpl10a
Rpl30
Rps7
Rpl19
Rpl13a
Rpl13
Rplp0
Rplp1
Rpl7a
Rpl11
Rpl12

D

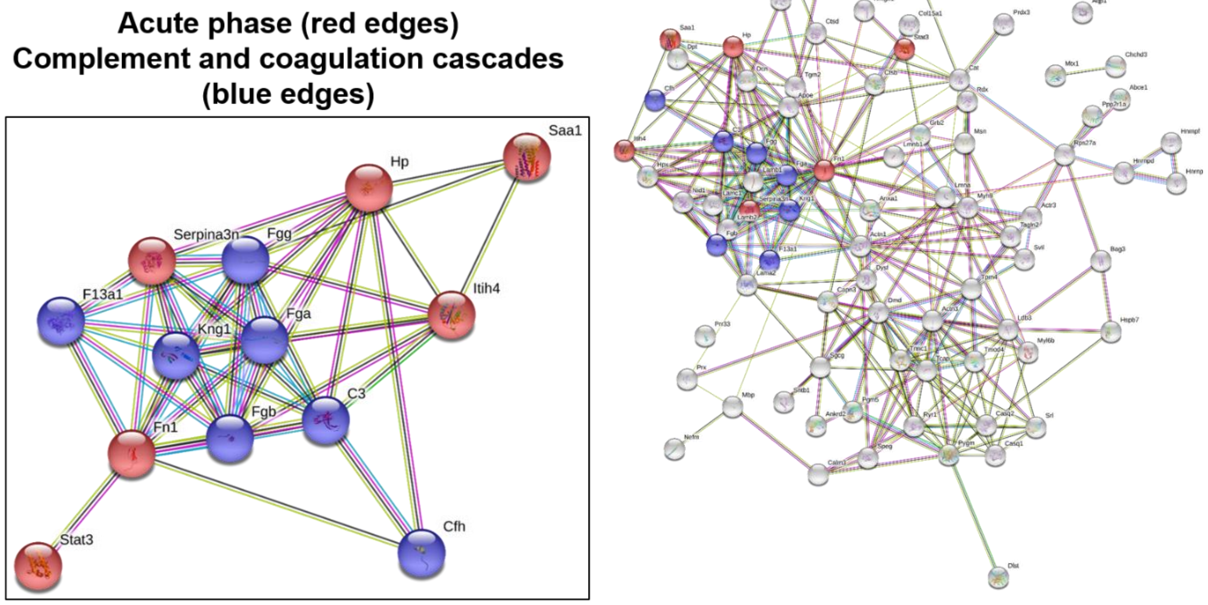
Acute phase (red edges)
Complement and coagulation cascades
(blue edges)
Saa1
Hp
Serpina3n
Fgg
F13a1
Itih4
Fga
Kng1
C3
Fgb
Fn1
Stat3
Cfh

Figure S1. Mitochondrial dysfunction, ribosome depletion and acute phase response take place in the skeletal muscle of C26 mice. STRING protein-protein interactions analysis obtained with DAPs in GC muscle during cancer cachexia: proteins associated with the enriched biological processes obtained with (A) the downregulated proteins (n = 120) and (B) the upregulated proteins in the SF (n = 117), (C) the downregulated proteins (n = 116) and (D) the upregulated proteins in the MF (n = 88) are highlighted in colors. The zoom insets show enriched items highlighted in Table 1 (see manuscript).

Figure S2

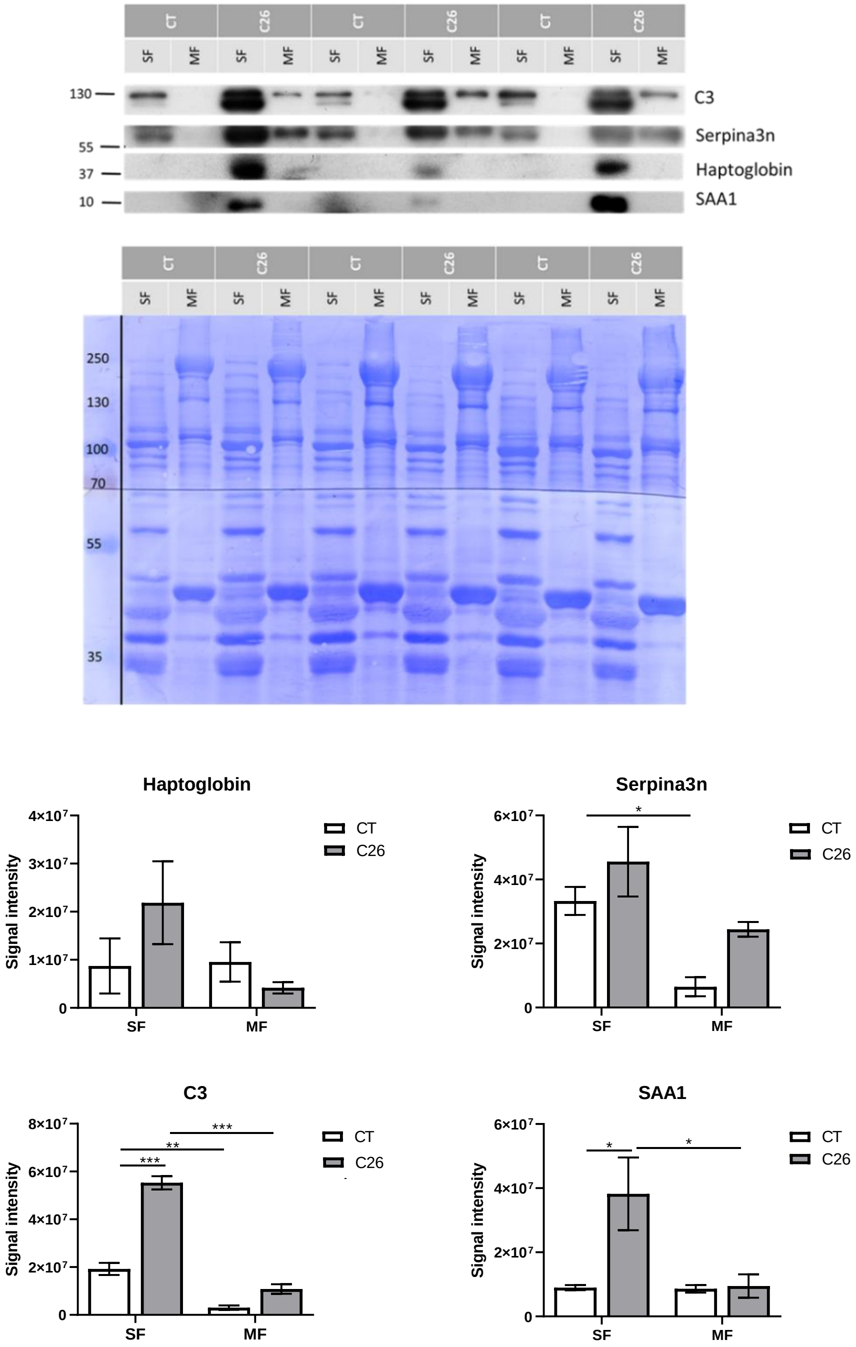

Figure S2: Hp, Serpina3n, C3 and SAA1 content in sarcoplasmic fraction (SF) and myofibrillar fraction (MF) of gastrocnemius (GC) from mice injected with C26 cells (C26) or vehicle (CT). Top: Protein levels measured by Western blot, Middle: Coomassie blue staining and Bottom: associated signal quantifications (n =3/group). 10 µg of protein were loaded by lane. Blots were cut during process to allow incubation with different primary antibodies. *$p < 0.05$, **$p < 0.01$ and ***$p < 0.001$.

Figure S3

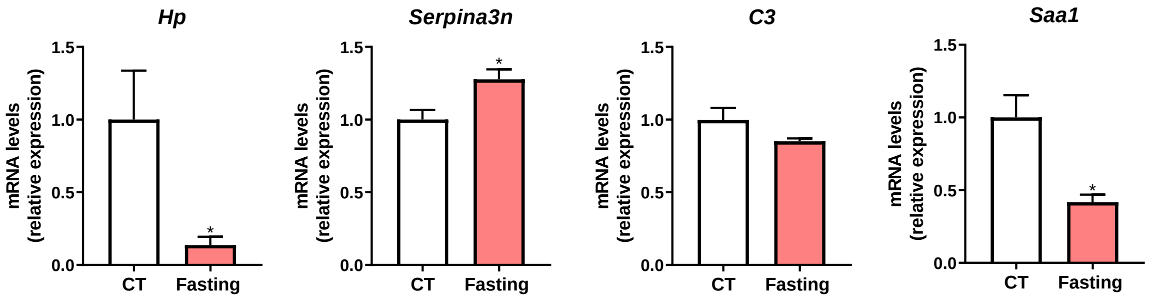

Figure S3: Effect of 48h fasting on muscle APR expression. Expression of acute phase reactants (APR) in GC muscle of CD2F1 control mice (CT) or 48h fasted mice (Fasting). (n=3/group). $*p < 0.05$.

Figure S4

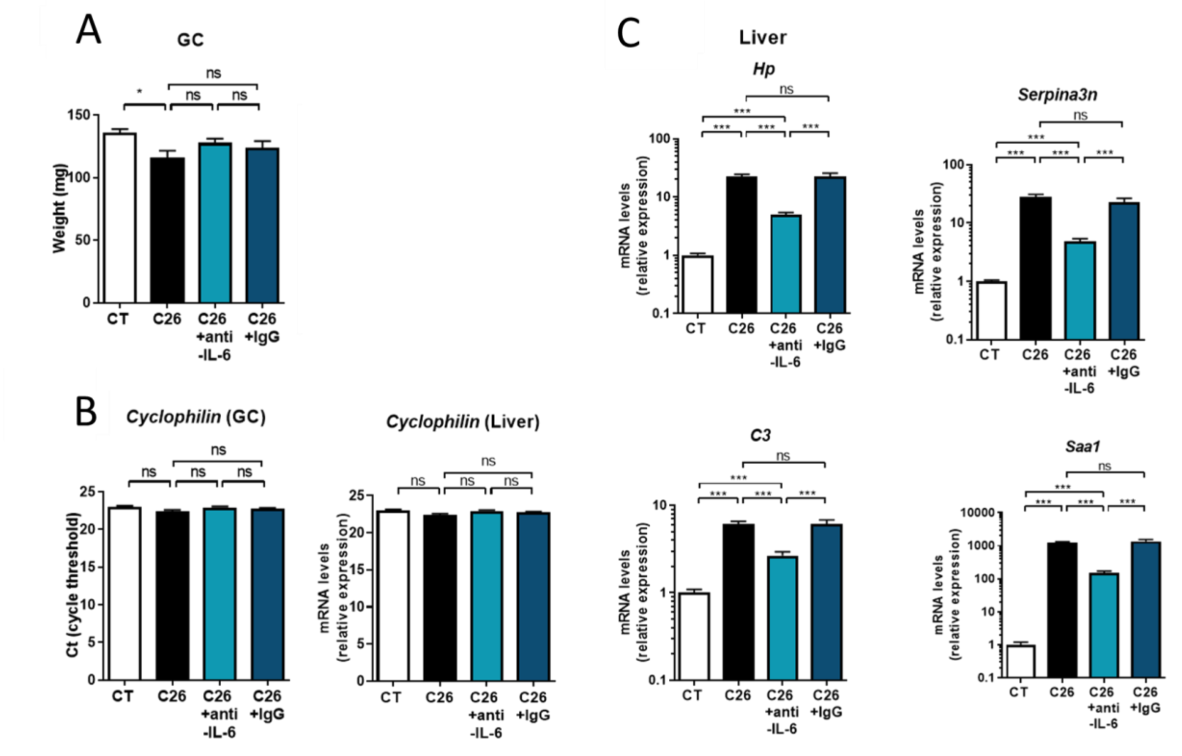

Figure S4: Anti-IL-6 antibody blunts muscle atrophy and prevents the induction of APR in the liver. (A) GC weight. (B) Housekeeping gene *cyclophilin* mRNA levels in the GC muscle and liver of mice injected with C26 cells alone (C26) or in combination with rat anti-murine IL-6 antibodies (C26 + anti-IL-6) or with rat IgG1 isotype control (C26 + IgG) or injected with vehicle alone (CT). (C) *Hp*, *Serpina3n*, *C3* and *Saa1* mRNA levels in liver of mice mice injected with C26 cells alone (C26) or in combination with rat anti-murine IL-6 antibodies (C26 + anti-IL-6) or with rat IgG1 isotype control (C26 + IgG) or injected with vehicle alone (CT) . (n=6-8/group). *p < 0.05 and ***p < 0.001.

Figure S5

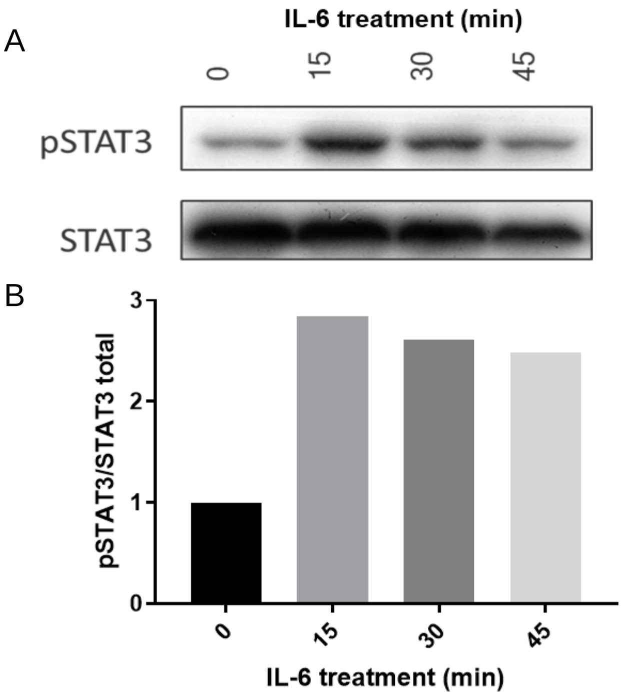

Figure S5. IL-6 enhances STAT3 phosphorylation in C2C12 cells. phospho-STAT3 and STAT3 levels (A) and relative quantifications (B) in C_2C_{12} myotubes exposed for 0, 15, 30 or 45 min to IL-6 25ng/ml.

Figure S6

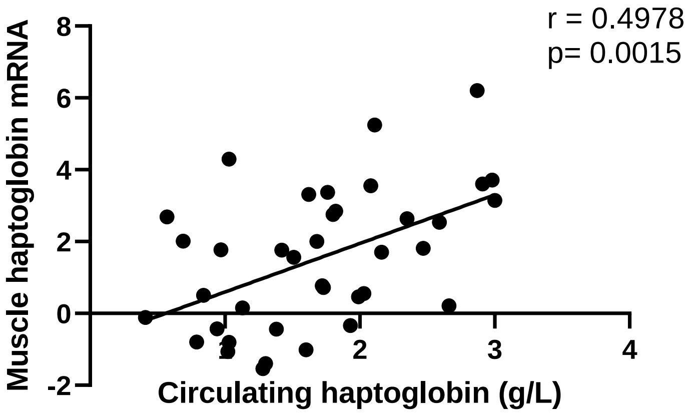

Figure S6: Correlation between circulating HP and HP muscle mRNA levels in cancer patients. Pearson correlation between circulating levels of HP and log2 transformed *HP* mRNA levels in muscle of cancer patients (Loumaye A et al., 2015).

Figure S7

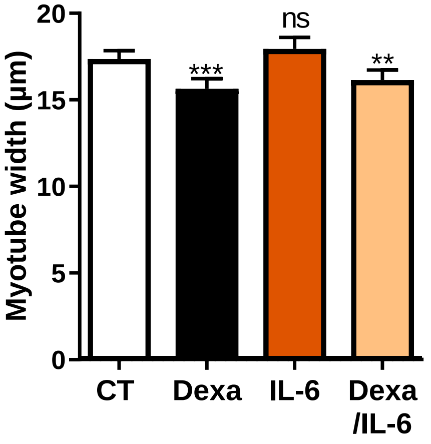

Figure S7. Myotube size in response to glucocorticoids and IL-6 *in vitro* treatments. C2C12 myotube size in response to IL-6 25ng/ml, Dexa (10^{-6} M) alone or in combination for 48h. n=3/group. $^{**}p < 0.01$ and $^{***}p < 0.001$.

Figure S8

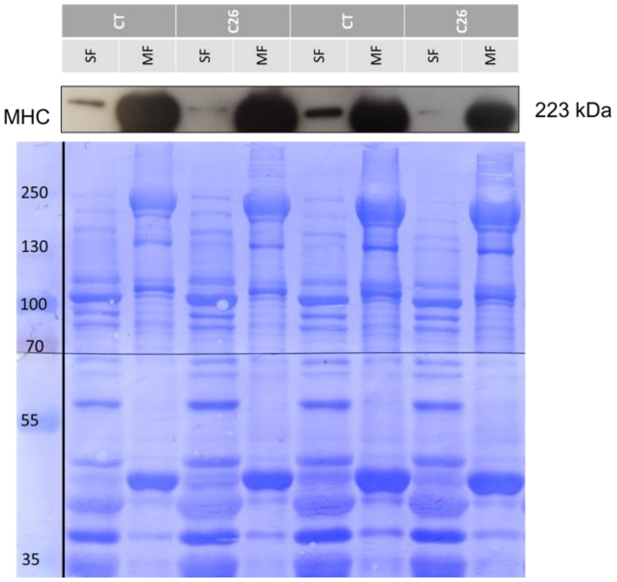

Figure S8: Enrichment of myosin heavy chains in the MF fraction. Top: Myosin heavy chains (MHC) content in SF and MF of gastrocnemius (GC) from mice injected with C26 cells (C26) or vehicle (CT). Western blot with anti-MHC (MF20 antibody; AB2147781; DSHB) targeting all MHC isoforms. 10 µg of protein were loaded by lane. Bottom: Coomassie blue staining.

Supplementary Tables legends

Table S1. List of the proteins identified and results of differential analyses comparing CT and C26 animals. See Supplementary Table S1. SF data (sheet #1) and MF data (sheet #2). NaN: Not a Number (corresponds to Non Valid Value).

Table S2. List of the proteins found differentially abundant (DAPs) between CT and C26 animals. See Supplementary Table S2. SF data (sheet #1) and MF data (sheet #2). C26 mice (n = 6) are in yellow and CT mice (n = 6) in Blue.

Table S3. Illustrations of the differential distributions of the selected peptides belonging to APR proteins of interest and measured by MRM at the circulating level in C26 and BaF3 animals and their respective CT. See Supplementary Table S3. Peptide sequence: targeted peptide for each protein. M: oxidized methionine; total: addition of peptide and its oxidized form. Results represent light/heavy peptide ratio. Light/heavy peptide corresponds to the total of the two most intense transitions. Results are expressed as mean ± SEM. Mann-Whitney test; /: no light peptide was detected.

Table S4. Technical validation on three replicates of the C26 plasma pool. In red and blue, the two most intense fragments (transitions) detected per peptide. The oxidized peptide shows M in bold font.

Table S5. List of primers sequences used for real-time quantitative PCR analysis.

Gene name	Transcript	Forward (5′-3′)	Reverse (5′-3′)
C3	Human	ATTTGCGAGGAGCAGGTCAA	GATGTGGCCTCCACGTTGTA
C3	Mouse	ACTGAAGGTCGTGCCAGAAG	GGGGTCACGATCAGGTGTTT
Fbxo32 (Atrogin-1)	Mouse	CCATCAGGAGAAGTGGATCTATGTT	GCTTCCCCCAAAGTGCAGTA
GAPDH	Human	CGCTGAGTACGTCGTGGAGTC	GCAGGAGGCATTGCTGATGA
Gapdh	Mouse	TGCACCACCAACTGCTTA	GGATGCAGGGATGATGTTC
HP	Human	GCTATGTGGAGCACTCGGTT	CAACAGCCTTATTTATCCACTGC
Hp	Mouse	TATCGCTGCCGACAGTTCTAC	CGTGGCGGGAGATCATCTTG
Murf1	Mouse	TGTCTGGAGGTCGTTTCCG	ATGCCGGTCCATGATCACTT
Ppid (Cyclophilin)	Mouse	TAAGCATGATCGGGAGGGTT	CGTCCAGATGAGGAGTCGGA
SAA1	Human	GAGCACACCAAGGAGTGATTTT	GAAAACCAGGCCCGTGAGA
Saa1/Saa2	Mouse	GCGAGCCTACACTGACATGA	TTTTCTCAGCAGCCCAGACT
SERPINA3	Human	GGCCTTTGCCACTGACTTTC	TCATGGGCACCATTACCCAC
Serpina3n	Mouse	GACCTGTCTGCAATCACAGGA	TTTGGGGTTGGCTATCTTGGC

PCR annealing temperature: 60°C

Table S6. - Characteristics of healthy subjects and cancer patients

	Healthy subjects	Cancer Patients	
	**Control**	**Non-Cachectic**	**Cachectic**
**N**	8	19	16
**Age (years)**	69 (61–77)	65 (44–83)	69 (40–95)
**Baseline BMI (kg/m^2)**	24 (21–31)	27 (22-34)	25 (20-39)
**Weight loss (%)**	0	0 (0–3)	7 (2–13)

BMI: body mass index.

Supplementary references

Lange, V., Picotti, P., Domon, B., & Aebersold, R. (2008). Selected reaction monitoring for quantitative proteomics: a tutorial. *Mol Syst Biol, 4,* 222. doi:10.1038/msb.2008.61
